# Ultrasound fundamentals and their clinical implications for interventional cytopathologists

**DOI:** 10.1111/cyt.13382

**Published:** 2024-04-18

**Authors:** David Lieu

**Affiliations:** ^1^ Department of Pathology UCLA Los Angeles California USA

**Keywords:** fine‐needle aspiration, instrumentation, interventional cytopathology, ultrasound fundamentals

## Abstract

**Objective:**

To describe the most important concepts in ultrasound physics that interventional cytopathologists need to understand in order to successfully perform ultrasound‐guided needle biopsies.

**Methods:**

Review of the literature.

**Results:**

A deep understanding of ultrasound physics and the mathematics supporting it are not necessary. The most important concepts are frequency, attenuation, overall gain, time‐gain compensation, focus, spatial resolution, temporal resolution and Doppler.

**Conclusion:**

By understanding these eight basic concepts of ultrasound physics and their clinical implications, interventional cytopathologists can faithfully reproduce the imaging findings of the radiologist and locate the target to precisely guide a needle for biopsy.

## Graphical abstract

Interventional cytopathologists need to know about ultrasound physics and instrumentation to produce an image that faithfully recreates the lesion discovered by the sonographer and diagnosed by the radiologist. A deep quantitative understanding of physics is not required. Knowledge of the basics of ultrasound and their clinical implications will help the interventionalist create a clear target for biopsy.

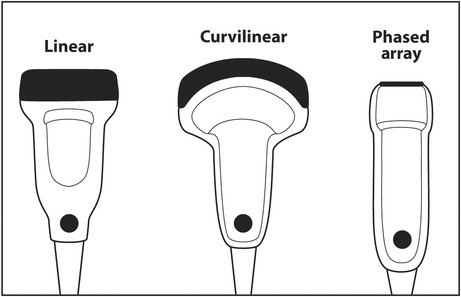

## 1 INTRODUCTION

What level of knowledge of ultrasound physics and instrumentation should healthcare professionals have in order to perform their job effectively? The answer depends largely on their role in the healthcare system. Some roles require a deep knowledge of ultrasound physics while others emphasize clinical applications. This paper will review the extent of knowledge of basic physics and instrumentation required of various healthcare professionals, compare ultrasound to other imaging modalities, outline the basic science underlying diagnostic ultrasound and link basic science to its clinical implications.

Medical physicists need a deep understanding of the basic science of ultrasound including the mathematics behind it. Research medical physicists and their engineering colleagues advance ultrasound by developing new methods of creating better images of the body to facilitate the detection of pathology. An example of this advance is the invention of CMUT (capacitive micromachined ultrasound transducer). The CMUT, such as the Butterfly iQ, allows an entire ultrasound machine to be contained in a hand‐held transducer when linked to an i‐Phone or i‐Pad to replace a bedside ultrasound machine weighing hundreds of pounds using PZT crystals to produce ultrasound.^1^ The image is not as clear as one from a large bedside ultrasound machine but the unit is cheap and portable.

Clinical medical physicists are experts in the application of ultrasound on phantoms to insure an ultrasound machine is functioning correctly in terms of spatial resolution, contrast, temporal resolution and bioeffects.^2^ They test ultrasound machines on a regular basis for quality assurance to verify that machines are functioning within manufacturer specifications. They help detect problems with a machine and order repairs or replacement when a machine is no longer functioning correctly. They also insure that machines meet regulatory requirements and teach radiology residents. However, they are not experts in interpreting clinical images of patients.

Radiologists are the face of ultrasound. They are the experts in interpreting ultrasound images. Since radiologists are physicians, they correlate the imaging findings with clinical findings and help clinicians make a diagnosis. They suggest the next step in the work‐up of a patient. They may perform interventional procedures under ultrasound guidance. Since their role is mostly clinical, a deep understanding of ultrasound physics is not required. Radiologists are usually not the persons doing the actual scans on patients or capturing the images. This role is performed by ultrasound technologists. Ultrasound technologists work as a team with radiologists. Technologists in the United States typically attend an ultrasound school that is part of a college or privately owned institution. The program is typically 2 years in duration. Students learn ultrasound physics, operation of the ultrasound machine, scan patients during clinical rotations and capture images of pathology. They must pass rigorous examinations on sonographic principles and instrumentation and in one or more clinical areas administered by the American Registry for Diagnostic Medical Sonography (ARDMS) to be employable. They are more expert in operating the ultrasound machine than radiologists.^3^ This teamwork is similar to radiologic technologists positioning patients and taking X‐rays while the images are interpreted by radiologists.

How much ultrasound physics and instrumentation should clinicians and interventional cytopathologists have in order to function effectively? While deep knowledge is admirable, it is not required. In particular, for an interventional procedure such as ultrasound‐guided fine‐needle aspiration (UG‐FNA) or ultrasound‐guided core‐needle biopsy (UG‐CNB), the operator must understand enough ultrasound fundamentals and operation of the ultrasound machine to re‐create the image of pathology previously captured by the sonographer and diagnosed by the radiologist. This will insure that the needle is guided into the correct lesion to obtain enough tissue to make an accurate pathologic diagnosis. If a poor image is created due to suboptimal settings on the ultrasound machine, UG‐FNA or UG‐CNB could result in sampling error due to a geographic miss. This paper will compare other imaging modalities to ultrasound and review basic ultrasound fundamentals and instrumentation with their clinical implications to assist interventional cytopathologists and others who perform ultrasound‐guided biopsies to create an image that is adequate to guide a needle into a target.

### 1.1 The spectrum of medical imaging

The oldest medical imaging modality is X‐ray. In 1895, Wilhelm Roentgen accidentally discovered X‐rays while working on an experiment with a Crookes vacuum tube.^4^ He showed bones in the hands of his wife on a photographic plate using the newly discovered X‐rays. The following year, Edward Frost was the first person to apply X‐rays for a medical purpose when he imaged a Colles fracture in a patient. For his discovery, Roentgen received the first‐ever awarded Nobel Prize in Physics in 1901.

X‐rays are produced by accelerating electrons from a cathode under high voltage in a vacuum tube impacting on a tungsten anode.^5^ The energetic electrons displace electrons from tungsten atoms and result in the production of X‐rays. These X‐rays can be passed through a patient and be detected by a chemical reaction on a photographic plate or, more recently, a detector connected to a computer, on the other side of the target. Dense tissue, such as bone, blocks the passage of X‐rays while soft tissue allows most of the X‐rays to pass through to the photographic plate or detector. X‐rays were first used to detect fractures. Modern advances in X‐ray technology include the production of less energetic, softer X‐rays and better contrast resolution that allows soft tissue to be imaged. The mammogram is an example of soft tissue imaging. By imaging at multiple angles and computer reconstruction of the output, a three‐dimensional image of the area scanned can be re‐created. These are the principles underlying CT scans and more recently, digital breast tomosynthesis (DBT). Imaging‐guided biopsy of non‐palpable masses can be done using CT guidance or DBT. These methods are not real time in that the needle is positioned at the edge of a mass or into a mass under imaging guidance. The needle is then advanced for the biopsy when the patient is taken out of the scanner.

While X‐ray and CT scan excelled in detecting bone lesions and some soft tissue lesions, the invention of magnetic resonance imaging (MRI) greatly improved visualization of soft tissue lesions.^6^ Unlike X‐rays that depend on their production by displacement of electrons in tungsten, MRI depends on the nuclei of hydrogen atoms in water, the most abundant compound in the human body, to produce an image. The nuclei of hydrogen atoms have an inherent spin at a frequency called the resonant frequency. The spinning protons have angular momentum. The patient is placed in a strong magnetic field. The magnetic field causes the spinning nuclei in hydrogen atoms to align at an angle relative to the magnetic field. A short radiofrequency pulse is introduced into the patient. When the hydrogen atom nucleus absorbs this energy at the resonant frequency, the spin flips to a higher energy state as dictated by the laws of quantum mechanics. When the pulse is turned off, the energy is released as a radiofrequency nuclear magnetic resonance signal (NMR). The time for the nuclei to return to the ground state and the frequency of the released energy can be measured in different planes and is a function of the types of atoms surrounding the hydrogen nuclei. These data are used to create T1 and T2 images for the radiologist. Anatomy is best displayed on T1 images. Fat and bone are bright white on T1. Most pathologic lesions contain water, such as high vascularity, ascites, and oedema. Water is bright white on T2. Therefore, most malignancies are white on T2. Imaging‐guided biopsy can be done using MRI. However, it is not real time. Also, gadolinium, which is white on T1, is typically injected into the patient as a contrast agent to better see a mass. Malignant masses are often hypervascular. The gadolinium washes out in a few minutes, so the biopsy must be done very quickly.

Positron emission tomography (PET) is a type of physiological scan that most commonly uses ^18^F‐labelled fluoro‐2‐deoxyglucose (^18^F‐FDG) as a substrate to produce an image.^7^ It is a radioactive analogue of glucose that is incorporated into tissues with high metabolism, such as tumours. The half‐life of radioactive fluorine‐18 is 110 min. It undergoes decay by positron (anti‐electron) emission. A positron is an anti‐matter particle. It interacts with a free matter electron and undergoes annihilation producing two 511 KeV photons travelling in opposite directions. Detectors surrounding the patient attached to a computer determine the spot of annihilation and produce an image. Knowing the activity of the area of interest, the amount of ^18^F‐FDG injected, and the weight of the patient, a standardized uptake value (SUV) can be calculated. The higher the SUV in a certain location, the greater the suspicion of malignancy.

Nuclear scintigraphy is another medical imaging modality that uses radioactive decay to produce an image.^8^ For example, in thyroid scintigraphy, radioactive technetium‐99 m as NaTcO_4_, which mimics iodine, is injected into a patient. The compound accumulates in the follicular cells of the thyroid gland but is not organized or attached to thyroglobulin. The radioactive technetium‐99 m has a half‐life of 6 h and decays by gamma‐ray emission. Gamma cameras placed around the thyroid gland detect the gamma rays and produce an image. The compound is rapidly excreted in the urine. ^99m^Tc is the most common radioisotope used in nuclear medicine imaging.

Ultrasound imaging is completely different from other imaging modalities in radiology. It produces images by transmitting high‐frequency sound into the body and waiting for echoes to return. The strength of the returning echoes and the time for the echoes to return are used to produce an image. This is similar to sonar used by submarines to detect objects underwater. It is also similar to radar, which uses radio waves and their returning echoes, to detect aircraft or missiles. Ultrasound has advantages over other imaging modalities. Unlike electromagnetic waves, such as X‐rays, ultrasound at the energy levels used in medical imaging does not have significant bioeffects on humans. X‐rays in high doses can cause DNA damage and lead to cancer or mutations. Unlike nuclear medicine scans or PET scans, no radioisotopes need to be created. The patient need not isolate temporally while a radioisotope decays. MRI requires a powerful magnet, typically in the range of 1–3 Tesla. One Tesla is 10,000 gauss. The earth's magnetic field has a strength 0.25–0.65 gauss. Participants in the scan room cannot have any metal on their person because the magnet can cause metallic objects such as pens, watches, coins and jewellery to fly across the room like a missile. Patients with ferromagnetic objects in the body, such as aneurysm clips, joint replacements, implanted cardiac defibrillators, pacemakers, etc., cannot have an MRI scan because these objects will heat during the scan. These precautions need not be taken during an ultrasound scan. Unlike other imaging modalities used to guide a biopsy, one of the greatest advantages of ultrasound‐guided biopsy is it is done in real time under direct visualization.

### 1.2 The nature of sound

Sound is a longitudinal mechanical wave that transmits energy through matter and causes a periodic oscillation in particle location, density and pressure.^9^ As a mechanical wave, it requires matter to travel. Matter can be gas, such as air, liquid, such as cyst fluid, or solid, such as human tissue. Unlike light, which is an electromagnetic wave, it cannot travel through a vacuum. In contrast to what is depicted in Hollywood movies, there is no sound in outer space because there is no matter in space between planets and stars. Sound is typically diagrammed by physicists as a sine wave that oscillates up and down and travels from left to right. As the ascending portion of the sine wave approaches molecules in the medium, the molecules first get closer together, which causes an increase in density and pressure. After the sine wave peaks and the wave descends, the molecules spread apart and the density and pressure decrease. This oscillation in particle position, density and pressure can occur very rapidly and is related to the frequency of sound. For example, in an ultrasound scan at 10 megahertz (MHz), which is a typical frequency used in scans of superficial structures, the oscillation will occur 10 million times per second. These changes in particle position, density and pressure are called the acoustic variables.

**FIGURE 1:**
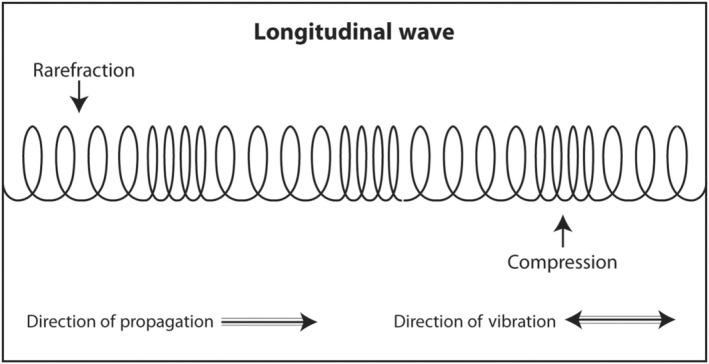
The sound waves transmitted into the body during diagnostic ultrasound and the returning echoes are longitudinal waves. They vibrate and travel in the same direction. Molecules in the medium are alternately compressed together and pulled apart as the sound waves passes.

Medical ultrasound produces longitudinal sound waves for transmission into the human body and receives sound echoes that are also longitudinal. That means that the sound waves are actually oscillating in the same direction of travel (Figure 1). This is similar to the 1960s ‘Slinky’ toy spring which oscillated in the same direction of travel as it ‘walked’ down the stairs. The reason that physicists draw sound as a sine wave oscillating up and down while travelling from left to right is because it is much easier to draw that way.

**FIGURE 2:**
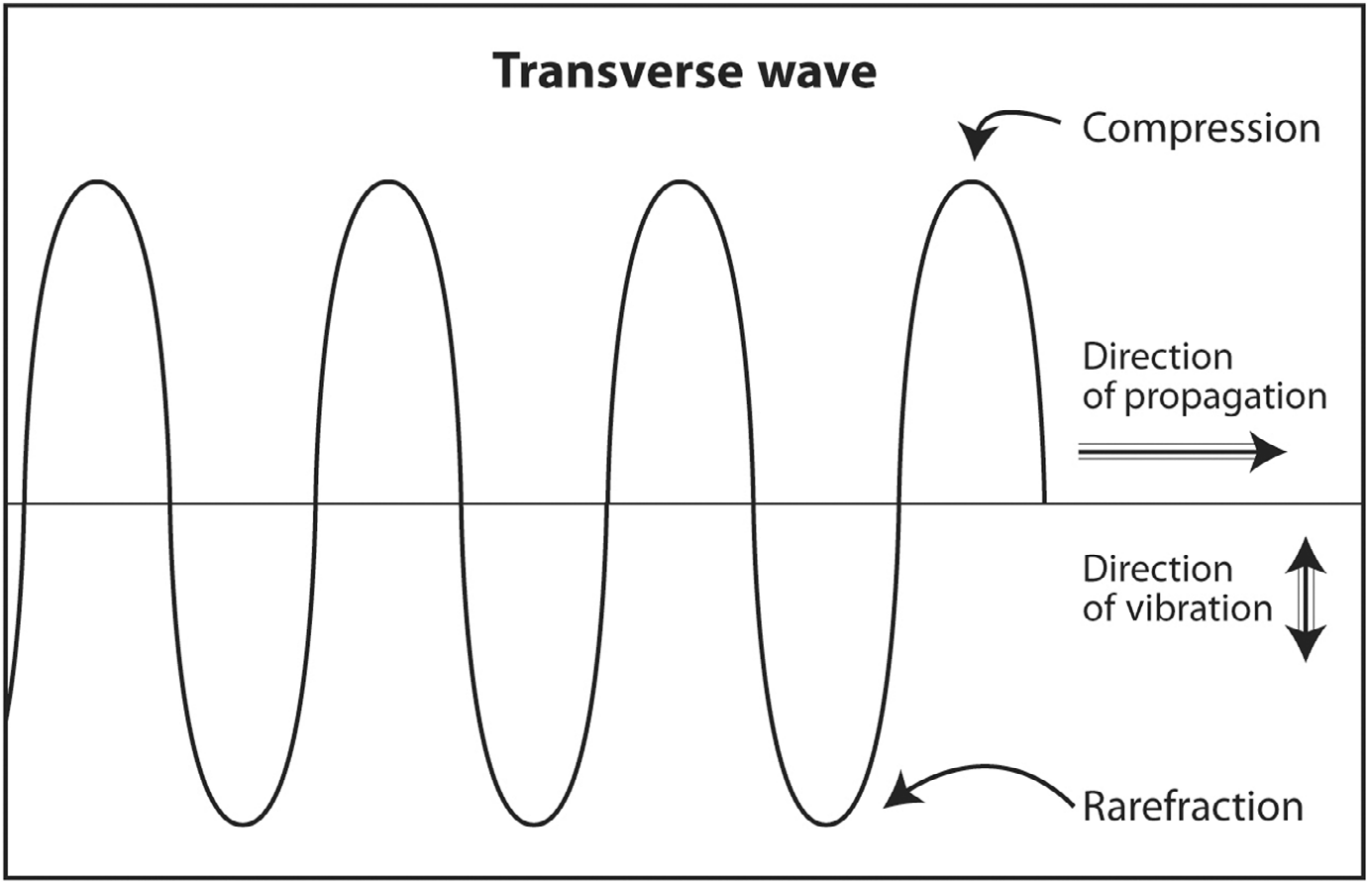
A transverse wave travels in a direction perpendicular to the direction of vibration. Transverse waves are also produced during diagnostic ultrasound. They travel much slower than longitudinal waves. They slightly degrade image quality on the ultrasound monitor similar to a blurred image if a subject moves while being photographed. In an advanced application called shear wave elastography, transverse waves can be used to determine the stiffness of an object.

Sound waves that oscillate in a direction perpendicular to the direction of travel are called transverse waves (Figure 2). Both longitudinal waves and transverse waves are produced as echoes that return from the body during an ultrasound scan. Transverse waves are not used to produce an ultrasound image. In fact, they slightly degrade an image. In an advanced application called shear wave elastography, they can be used to determine the stiffness of a mass. Stiff masses are more likely to be malignant than soft ones. An example of the production of a transverse wave is dropping a rock into a lake. Water waves travel concentrically away from the point of impact but oscillate up and down.

Why is sound depicted as a sine wave? There are other waves that are periodic. Why is sound not depicted as a square wave, triangular wave, elliptical wave or other periodic wave? The answer lies in the quantitative definition of sound. Physicists describe sound as a second‐order partial differential equation with four variables: *x*, *y* and *z* (spatial or Cartesian coordinates) and *t* (time). The sine wave is a solution to that equation. Sound in real life is much more complicated than a simple sine wave. An example is music. It consists of many sound waves with different frequencies and amplitudes. But complex sound can be broken down and represented as a sum of sine and cosine waves that are solutions to the second‐order partial differential equation of sound. This is called Fourier analysis.

### 1.3 Acoustic parameters

Once the existence of sound is established either qualitatively as a mechanical wave travelling through matter or quantitatively as a sum of sine and cosine waves, four parameters are required to describe everything about this sound: frequency, power, propagation speed and wavelength. With the knowledge of the four acoustic parameters, the initial location in space and time at *t* = 0 and the Fourier transform of the sound, physicists can predict the location and phase of sound in the medium through which is travelling at time *t* > 0. Such detailed knowledge of mathematics is not required for interventional cytopathologists and others who perform ultrasound‐guided needle biopsies. However, basic knowledge of the clinical implications of the acoustic parameters will enhance the operator's understanding of what is appearing on the screen and his or her ability to adjust the image to visualize a target for biopsy.

### 1.4 Frequency

**FIGURE 3:**
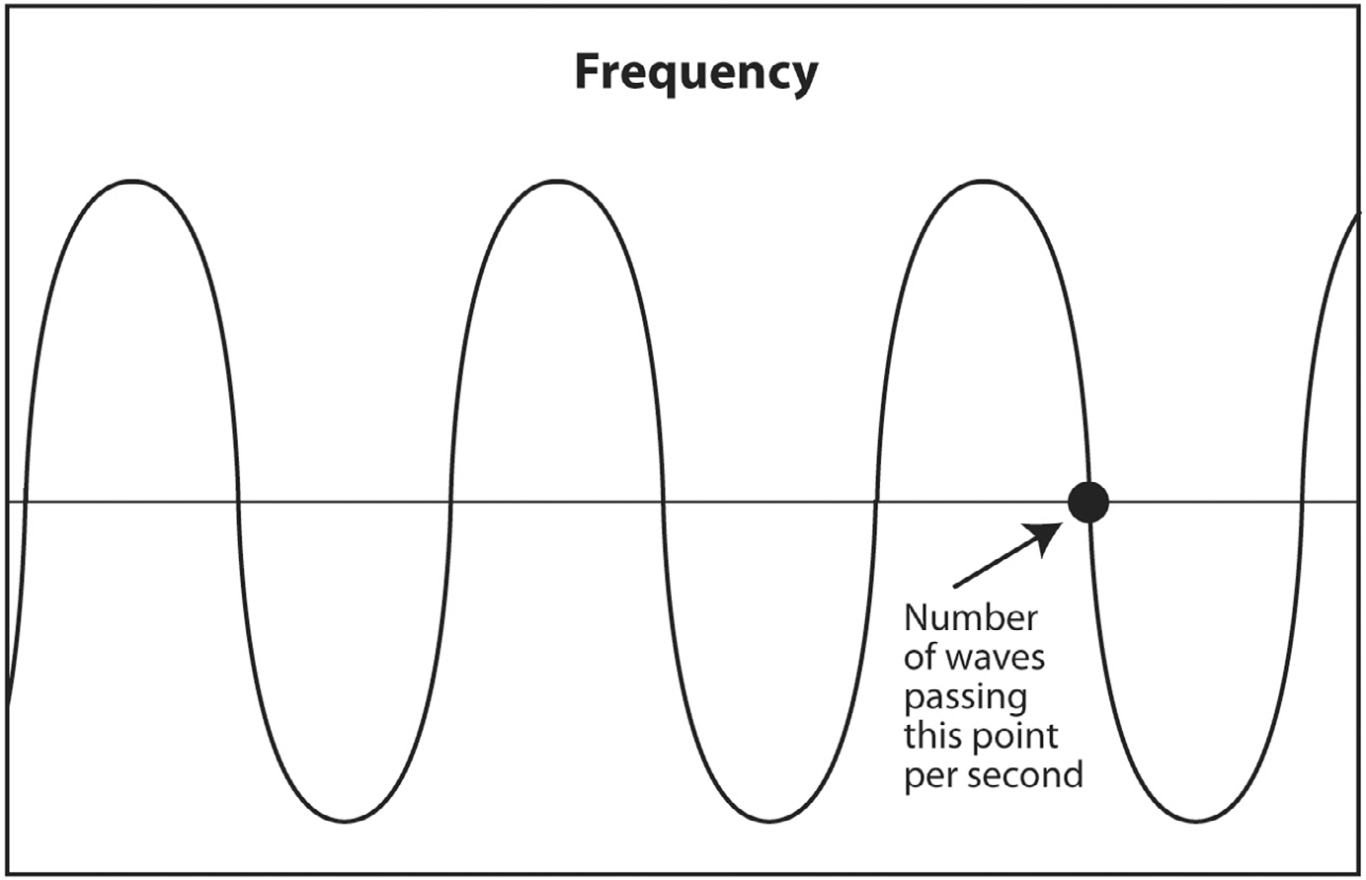
Frequency is arguably the most important acoustic parameter in diagnostic ultrasound. High‐frequency sound results in the best axial (vertical axis) resolution. Unfortunately, high‐frequency sound does not penetrate very deeply into the body. The clinical implication is to use the highest frequency sound consistent with the sound penetrating deep enough to adequately view the object of interest.

The first acoustic parameter that describes sound is frequency. Frequency is probably the most important parameter to be understood by the interventionalist. The frequency of a sound wave is the number of complete waves that pass a point in space in 1 s (Figure 3). The units are sec^−1^ or hertz (Hz). The frequency range of audible sound is 20–20,000 Hz. Sound that is below the lower limit of audible range (<20 Hz) is called infrasound. It has no application in diagnostic medical ultrasound. Sound with a frequency above the upper limit of audible range (>20,000 Hz) is called ultrasound. Certain frequencies of ultrasound are used in diagnostic medical ultrasound. Depending on the anatomic area being scanned, typical frequencies used to scan a patient are 2.5–24 MHz (megahertz or millions of hertz).

The frequency of sound is determined by the source. The thinner the crystal producing the sound, the higher the frequency of sound it will produce. An everyday example of this is when a spoon strikes two different glasses of different thicknesses. The thinner glass will produce a higher frequency sound. To produce sound with a frequency of millions of hertz, the crystals in an ultrasound transducer must be very thin. As sound passes from one medium to another, such as passing through, skin, fat, muscle, a mass and bone during a diagnostic ultrasound scan, the frequency does not change. The importance of frequency is twofold. The higher the frequency used in a scan, the higher (better) the axial resolution.^10^ The axial plane is the plane in which the beam is travelling into the body. It corresponds to the vertical axis (up and down) on the ultrasound monitor. A high‐frequency scan will be able to distinguish two objects that are close to each other in the axial plane. A low‐frequency scan will result in the two objects appearing as a single larger object. In a sense, a high‐frequency scan is like a probe with a fine tool, whereas a low‐frequency scan is like a probe with a coarse tool. The fine tool will be able to distinguish two objects close to each other as two separate objects rather than as one large one.

If high‐frequency results in better axial resolution, why not use extremely high frequencies to detect tiny tumours at the earliest possible stage? High‐frequency sound is a two‐edged sword. High‐frequency sound is absorbed (attenuated) by tissue very quickly. In other words, very high‐frequency sound does not penetrate very deeply into the body. It is converted to heat at superficial depths. The reason for the inability of high‐frequency sound to penetrate deeply is the first law of thermodynamics, also known as conservation of energy. Ultrasound that is transmitted into the body has four possible outcomes: conversion to heat, transmitted deeper into the body, scattered in multiple directions or reflected at tissue interfaces as an echo back to the transducer. High‐frequency sound causes molecules in the medium to vibrate back and forth very quickly and produce heat. The energy of the sound wave is converted to heat and little energy is left in the wave to travel deeper, scatter or reflect back to the transducer as an echo. Lower frequency sound causes less vibration of molecules and less conversion to heat. Thus, there is more energy in the sound wave to transmit deeper into the body, scatter or reflect back to the transducer as an echo. The chosen frequency for an ultrasound scan is a balance between the best attainable axial resolution versus adequate penetration to visualize the object of interest. The interventionalist should choose the highest frequency consistently that adequately images the object of interest. Superficial objects, such as thyroid, head and neck, salivary glands, breast, axilla and subcutaneous tissue, are typically scanned at 10–18 MHz. Deeper objects, such as abdomen and pelvis, are scanned at 5 MHz. A very deep object, such as the heart, is scanned at 2.5 MHz. The scan should start using a transducer with the highest frequency. If the object of interest is deep and appears too dark on the monitor, the gain (amplification) should be increased to increase its brightness. If it is still too dark, a lower frequency should be selected. If the object of interest is still too dark after maximizing gain and using the lowest frequency setting on a particular transducer, a different transducer with a lower frequency output should be selected.

### 1.5 Power

**FIGURE 4:**
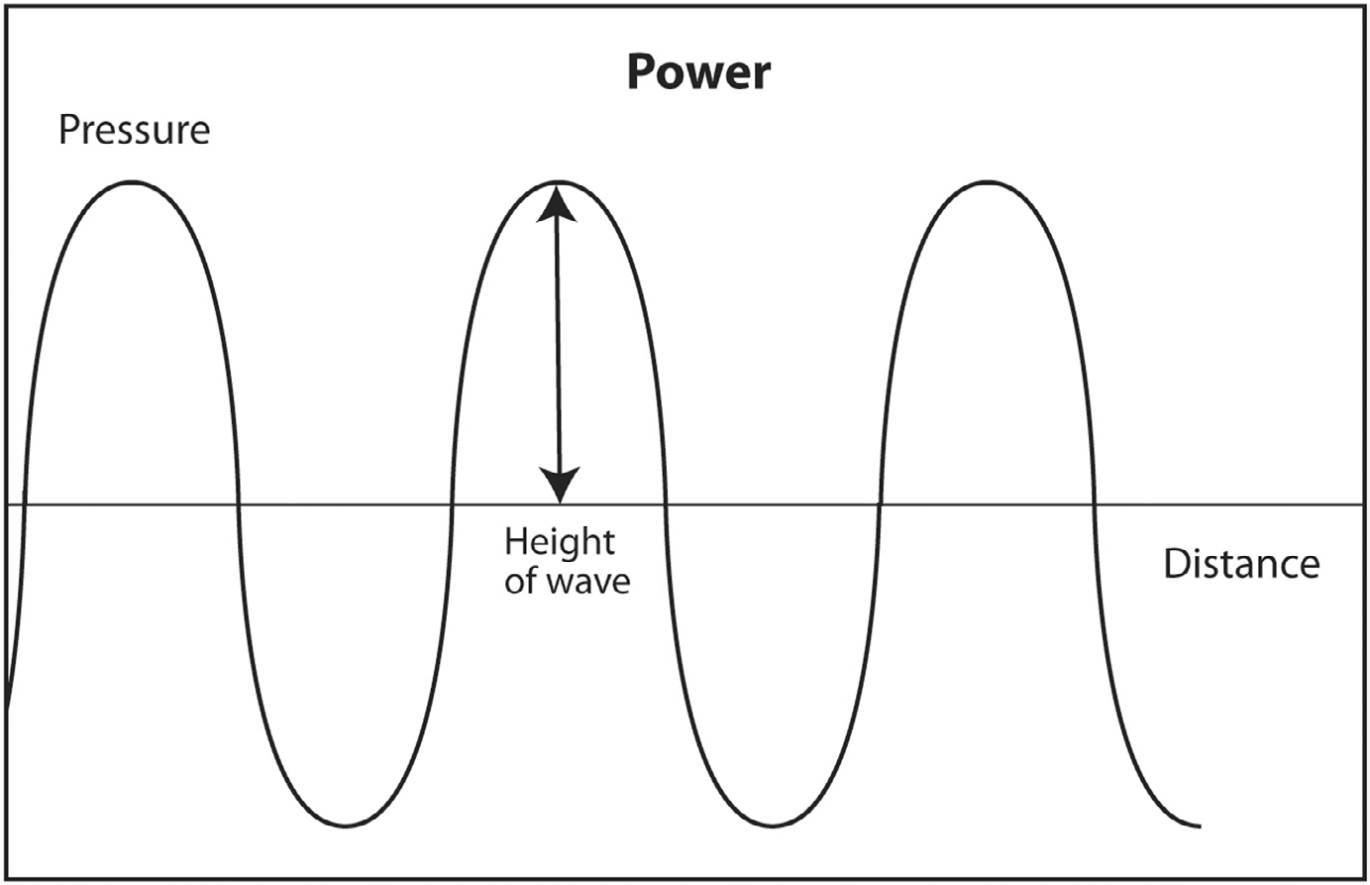
The energy of a sound wave is the height of the sine wave. For audible sound, it is the loudness of sound. If high powered sound is transmitted into the body, the returning echoes will enough be strong and create a brighter image. High acoustic power results in high signal‐to‐noise ratio of returning echoes and creates better images. However, the ALARA principle states that the lowest energy and shortest scan time consistent with obtaining a complete study should be used to minimize patient exposure to sound radiation. In the United States, ultrasound machines are regulated by the FDA and are safe at all levels of acoustic output. The only caution in using high acoustic power for imaging is in foetal scans or retinal scans. These are not likely to be performed by interventional cytopathologists.

The second acoustic parameter that describes sound is power. This parameter is perhaps the easiest to understand. Power is the strength of the sound wave. For audible sound, it is the loudness of the sound. Mathematically, it is represented as the height of the sine wave (Figure 4). It is measured in watts. It is determined by the source of the ultrasound, that is, the ultrasound machine, and can be adjusted by the interventionalist. As previously discussed, most of the energy of ultrasound transmitted into the human body during a diagnostic scan is converted into heat. It fact, there was fear during the earliest experiments with ultrasound that the sound would cook an organ like a microwave oven instead of obtaining useful anatomic images. Fortunately, that did not happen. The energy of diagnostic ultrasound is about 4–90 mW. This is not enough energy to cause damage to the human body. Ultrasound is a type of radiation that does not cause DNA damage. As a comparison, the energy output of a presentation laser is <5 mW. Since ultrasound is generally believed to be safe, ultrasound technologists in most states in the United States are not required to be licensed by the government in order to work. In contrast, X‐ray technologists must be licensed to work to insure a safe patient experience because X‐rays in high doses can be dangerous and cause DNA damage. Currently, CT scans must be done in a manner to minimize patient exposure to X‐rays consistent with obtaining an adequate study. In the United States, ultrasound machines are regulated by the Food and Drug Administration (FDA). All such machines are safe if used for their intended purpose.

Just because ultrasound is generally safe for humans does not mean it should be used indiscriminately. There is a principle called ALARA, which stands for ‘as low as reasonably achievable’, which governs how ultrasound and other imaging modalities should be used to maximize patient safety.^11^ Basically, ALARA states that patient exposure to radiation should be avoided if it has no direct benefit to the patient. Clinically, this means that diagnostic ultrasound should only be performed when indicated for diagnostic purposes. For example, an obstetric scan in a pregnant patient to see the foetus move and take pictures without a clinical indication would violate the ALARA principle. Patient exposure to radiation must be minimized consistent with obtaining a complete scan. This means the energy output used for a diagnostic ultrasound and time of exposure to the sound should be minimized as long as a complete scan can be obtained.

Interventionalists can adjust the power output of ultrasound. It is typically represented as a percentage of maximum power output and displayed on the monitor. What power output should be chosen for diagnostic ultrasound? As previously discussed, most ultrasound transmitted into the human body is rapidly converted to heat. This is especially true for high‐frequency sound as molecules in the medium oscillate rapidly. Since most of the energy is converted to heat, the returning echoes from interfaces between different tissues are very weak. The ultrasound machine amplifies (gain) the returning echoes into strong electrical signals that are then used to create an image on the monitor. If the transmitted sound is strong, the returning echo will be strong. If the transmitted sound is weak, the returning echo will also be weak but more gain can be used to amplify the signal and produce an image. According to the ALARA principle, lower output energy and more gain should be used to minimize patient exposure to ultrasound. But as previously discussed, FDA‐regulated ultrasound machines in the United States are safe at all levels of power output. In reality, ultrasound scans are typically done at maximum (100%) power output and lower gain settings. The reason is that noise in returning echoes (non‐useful background echoes that degrade the image) is constant at all levels of acoustic power transmitted into the body. But useful echoes returning to the ultrasound machine that convey useful information increase as the power of transmitted sound increases. This means that the signal‐to‐noise ratio (S/N) increases as the power of the transmitted ultrasound increases. Note that as S increases in the numerator and N stays constant in the denominator, the S/N ratio increases. Clinically, this results in a clearer image on the monitor. In general, interventionists should use 100% power output in most scans and adjust the gain to produce a readable image.

Ultrasound machines display the energy transmitted into the body during a scan. Power is measured in watts but this unit is more useful to physicists than to interventionalists. What is important is the potential bioeffects during a scan. These bioeffects are displayed on the screen as thermal index (TI) and mechanical index (MI).^12^, ^13^

TI is easier to understand. Mathematically, it is the acoustic power in the region in the body of maximum heating divided by the power needed to heat the tissue in that area by 1°C. More simply, it is approximately the maximum amount of tissue heating in the area that receives the most acoustic energy, which is at or just superficial to the focal point in the body where the sound beam is narrowest. Heating of tissues 2 degrees or less has no harmful bioeffects. Therefore, acoustic power output should be adjusted to keep TI ≤2 in most scans. It is typically ≤2 even at 100% power output during B‐mode (imaging mode for anatomy) scans.

MI measures the potential adverse effect of tissue destruction caused by mechanical energy. This mechanical effect is caused by microbubbles of gas in the body that rapidly expand, contract and burst as ultrasound is directed into the body. This bursting of microbubbles can cause mechanical damage to tissue in the immediate vicinity of the bubble. Mathematically, it is the peak rarefactional pressure of sound measured in megapascals (pressure at the bottom of the sine wave) divided by the square root of the frequency of the sound measured in megahertz. The math is not important but the ratio is important. According to the American Institute for Ultrasound in Medicine (AIUM), MI should be ≤1.9 to be safe. It is typically ≤1.9 even at 100% acoustic power output during B‐mode scans.

### 1.6 Propagation speed

**FIGURE 5:**
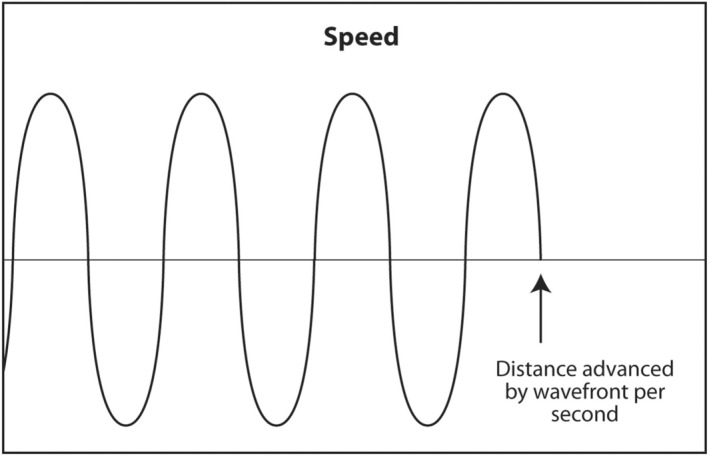
Propagation speed of sound is solely determined by the medium through which sound is travelling. It is independent of the frequency and power of sound. In general, sound travels fastest through stiff, low‐density mediums and slowest through soft, high‐density mediums. For example, sound travels fastest through solids, slower through liquids and slowest through gases. Although stiff mediums like solids also tend to be dense, stiffness usually increases much faster than density.

The propagation speed of sound through a medium is the third acoustic parameter that describes sound (Figure 5).^14^ It is determined solely by the characteristics of the medium. Changing the frequency of sound or its power will not change its speed. Sound travels fast in mediums that are stiff and low density and slow in mediums that are soft and dense. For example, sound travels about 330 m/s through air, 1540 m/s through human soft tissue and 4500 m/s through bone. Stiffness increases from air to soft tissue to bone. Density also increases in the same sequence but stiffness increases much faster than density. Therefore, the speed of sound increases in the same manner.

**FIGURE 6:**
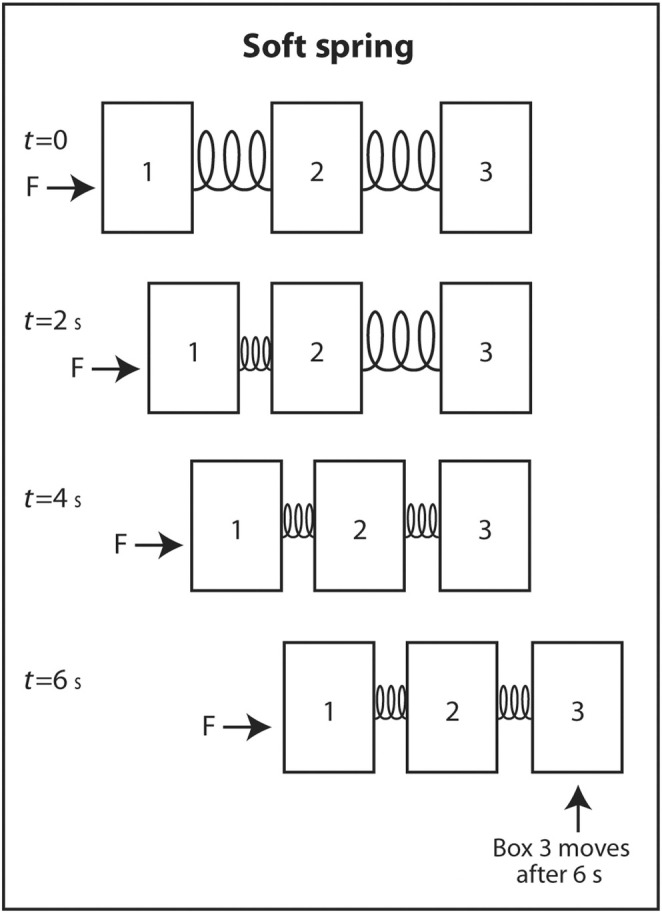
Three identical boxes are connected by springs that are not very stiff. If a horizontal force is applied to move the boxes starting at box 1, it takes a long time for each spring to be compressed enough before the next box moves. Therefore, it takes a long time before the wave front at box 3 finally moves. Similarly, sound travels slowly in mediums that are not stiff.

**FIGURE 7:**
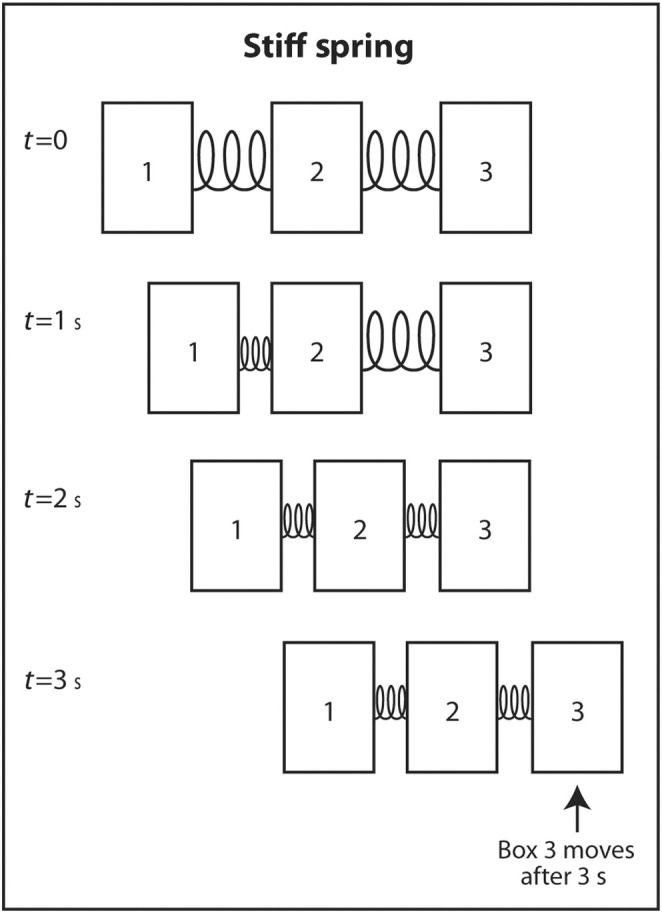
Three identical boxes are connected by stiff springs. If a horizontal force is applied to move the boxes starting at box 1, it takes a short time for each spring to be compressed enough before the next box moves. Therefore, it takes a short time before the wave front at box 3 finally moves. Similarly, sound travels faster through mediums that are stiff.

The simplest explanation for why sound travels faster in stiff low‐density mediums is as follows. Imagine three identical rectangular boxes on the floor in a row. Box 1 is connected to box 2 by a metal spring and box 2 is connected to box 3 by an identical metal spring. A horizontal force is applied on box 1 to move the three boxes in the same direction. As box 1 moves forward, box 2 does not move until the spring connecting box 1 to box 2 is sufficiently compressed. When the interconnecting spring is sufficiently compressed, box 2 starts moving forward. Box 3 does not move until the spring connecting box 2 to box 3 is sufficiently compressed. Once this spring is sufficiently compressed, box 3 starts moving forward. If the two springs are not stiff (easily compressible), it takes longer for the springs to be compressed sufficiently for the next box to move forward (Figure 6). In contrast, if the springs are very stiff, they only have to compress a little before the next box starts moving forward (Figure 7). This explains why sound travels faster through stiff mediums than compressible mediums. If the boxes are heavy, they will be dense. If they are light, they will have low density. Therefore, the boxes will move slower if the boxes are heavy than if they are light. The same analogy applies to the speed of sound through a dense medium versus a non‐dense medium. It travels faster through a non‐dense medium.

The clinical significance of the speed of sound is that certain assumptions are made to calculate the depth and dimensions of an object of interest in an ultrasound scan. Ultrasound machines assume that sound transmitted in the human body travels 1540 m/s through soft tissue and the returning echoes travel at the same speed. In different more useful units, 1540 m/s is the same as 1.54 mm/μs. If the speed of sound is different than this assumption, it will cause a misregistration error of the object on the monitor. The error is usually small because the speed of sound is only slightly different in fat, muscle and tumours and is usually not clinically relevant for the interventionalist.

**FIGURE 8:**
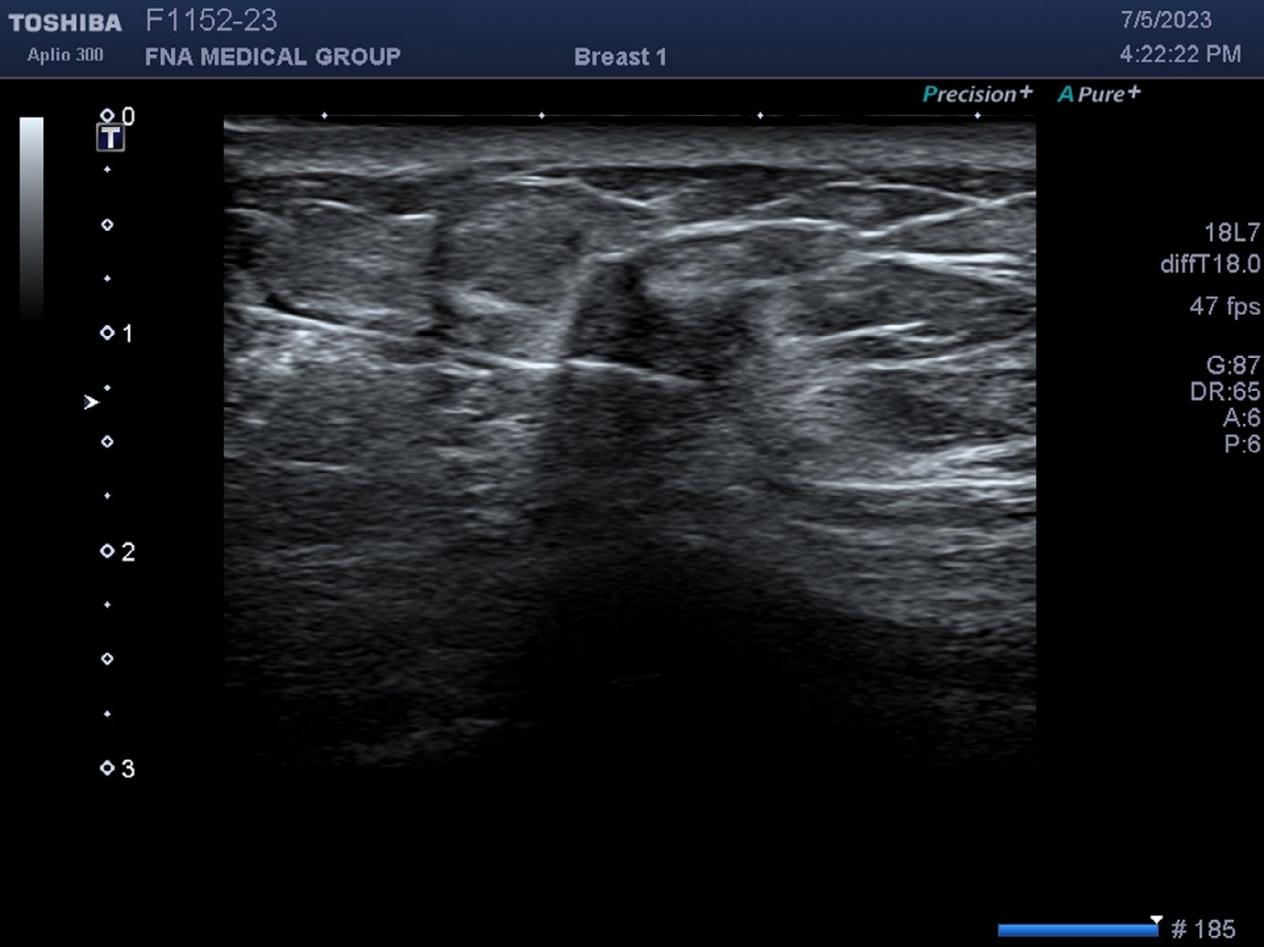
An FNA needle is entering a malignant breast mass. It appears to be broken or bent slightly upward at the entry point. This is a speed propagation artefact. The needle is not broken or bent. The malignant mass is stiffer than the surround normal breast. Sound entering the mass and the subsequent returning echoes from the needle reach the transducer sooner than sound from the surrounding normal breast. The machine assumes the early returning echoes from the needle within the mass are closer to the skin and misregisters them upward.

There are some misregistration errors that are interesting. Malignant neoplasms are usually stiffer than surrounding normal tissue due to desmoplasia. Therefore, sound travels faster through these tumours than through normal tissue. When an FNA needle enters the mass, the needle may appear to be bent upward at the point of entry into the mass because sound travels faster through the tumour than through normal surrounding tissue and the echoes returning from the needle arrive at the transducer sooner (Figure 8). The machine assumes that these earlier returning echoes from the needle in the mass must be closer to the skin than the part of the needle that is in normal tissue. This is called speed propagation artefact or, more colourfully, the bayonet sign. It is not always seen when an FNA needle enters a mass depending on the speed of sound through the different mediums. However, when it is seen, it proves that the needle is definitely in the mass.

**FIGURE 9:**
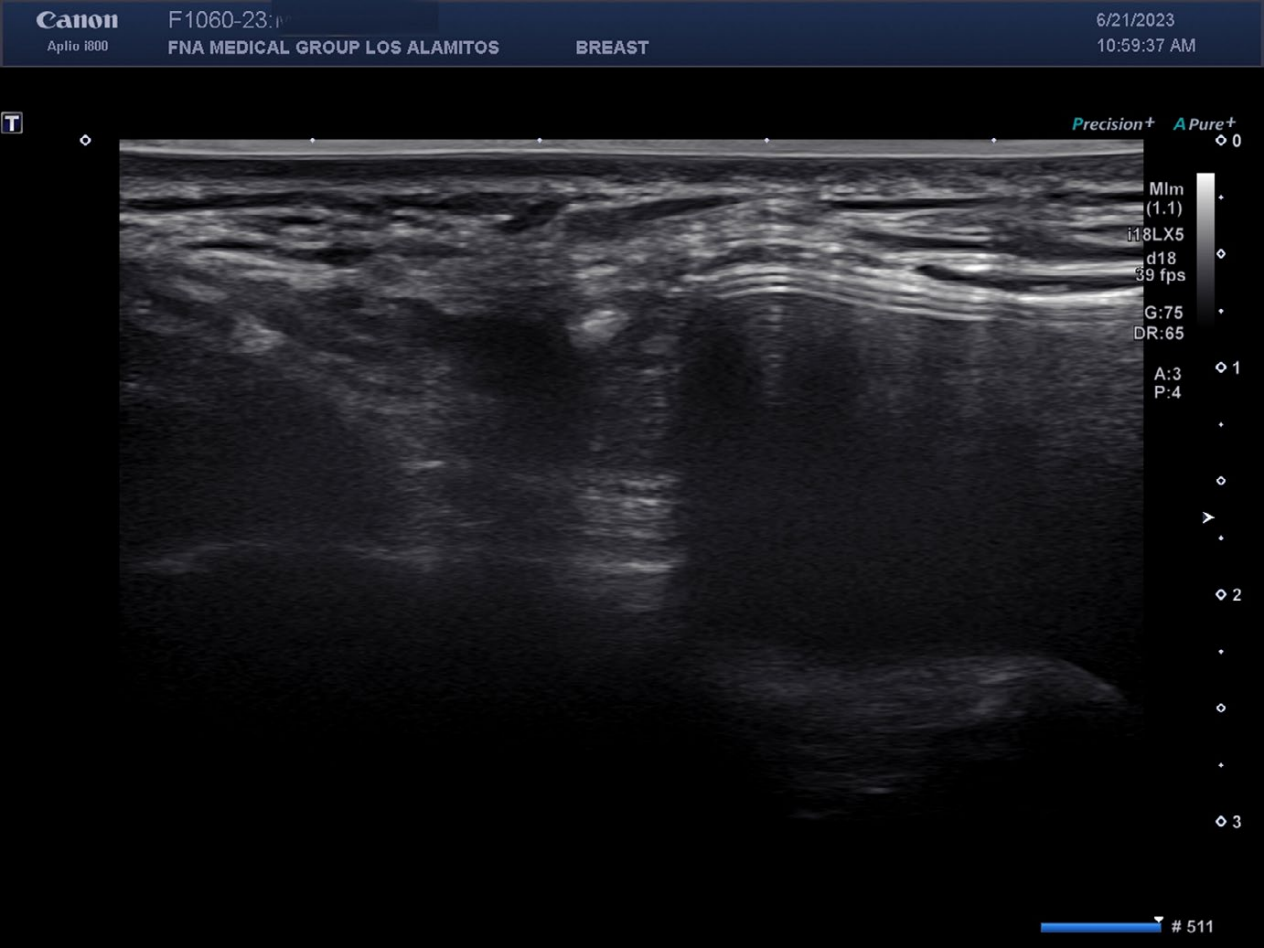
This patient has a silicone breast implant. It appears anechoic (black) on the right‐hand side of the screen. The pectoralis muscle and pleura at the left‐hand edge of the implant appear to suddenly drop deeper beneath the implant. Sound travels slower through silicone than through normal breast tissue. Therefore, the ultrasound machine assumes that the pectoralis muscle and pleura deep to the implant are much deeper than they really are. This speed propagation artefact results in a step‐off at the edge of the implant.

Another interesting application of speed propagation artefact is in breast imaging. There are two basic types of breast implants: saline and silicone. While silicone implants feel more natural than saline implants, they cannot always be distinguished by palpation. On mammogram, they can easily be distinguished because saline implants are translucent while silicone implants are opaque. On ultrasound, both types of implants are anechoic (black) and are indistinguishable. However, sound travels much slower through silicone than saline. Therefore, echoes returning to the transducer from tissue posterior to the implant will arrive later than echoes from tissue at the same depth that did not pass through the implant. At the edge of the implant, there will appear to be a step off of tissue posterior to the implant (Figure 9). The tissue is actually continuous but the abrupt step‐off is an artefact because the machine assumes all sound travels at the same speed no matter what tissue it went through.

### 1.7 Wavelength

**FIGURE 10:**
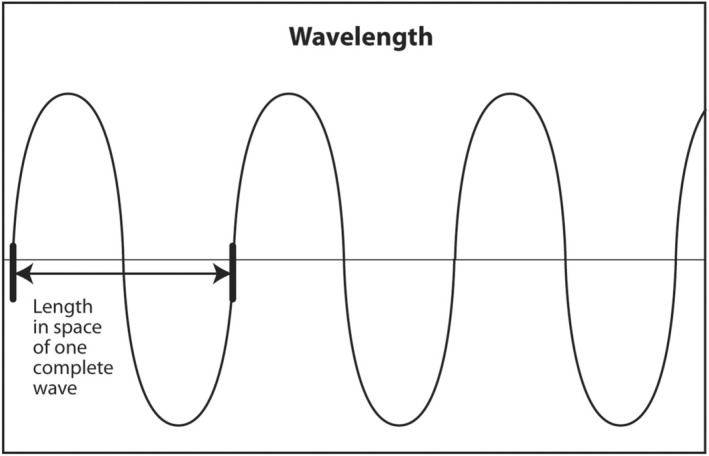
Wavelength is the length in space of one complete sine wave of sound. It is determined by the source (frequency of sound produced by the ultrasound machine) and the medium. If the speed of sound is constant, wavelength and frequency and inversely proportional. Wavelength and its related entity, spatial pulse length, are key determinants of axial resolution.

The wavelength of sound is the fourth acoustic parameter. Unlike the other three parameters, it is not independent. Wavelength is determined both by frequency, which depends on the source, and speed, which depends on the medium. Therefore, the wavelength of sound depends on the source and the medium. Physically, wavelength is the length in meters (or other units of length) of a single complete sine wave of sound. It can be calculated by knowing the frequency of sound and the speed of sound through a medium (Figure 10).

Suppose we wish to calculate the length of a boxcar in a moving train. Each boxcar is identical in length and we will ignore the length of the coupling between boxcars. The train is moving forward at 30 m/s (speed). At any fixed point along the tracks, two boxcars are seen passing per second (frequency). Then, each boxcar must be 30/2 = 15 m long. Suppose in a slightly different scenario, the train is still moving 30 m/s (speed). But now we see that there are 1.5 boxcars passing any given point on the tracks per second (frequency). Intuitively, if there are fewer boxcars passing per second and the speed is still the same, each boxcar must be longer. The calculation is 30/1.5 = 20 m long. Intuition and mathematics are congruent.

In general, the equation relating wavelength, frequency and speed is (*λ*) (*υ*) = *c* where *λ* = wavelength, *υ* = frequency and *c* = speed. Frequency of sound from a transducer does not change when it passes from one medium or tissue into another. The speed of sound depends only on the medium and averages 1.54 mm/μs in soft tissue. It changes only a little between various types of soft tissue. This means c is essentially constant in soft tissue. If frequency is changed by the touch of a button on the ultrasound machine or by using a different transducer and speed is essentially the same, wavelength must change in order for the equation to hold. In other words, if frequency is increased, wavelength must decrease. Wavelength and frequency are inversely proportional when speed is constant.

The implication of the clinical importance of wavelength is that when the sonographer or interventionalist changes frequency by pushing a button or changing to a different transducer, he or she is changing the wavelength. The wavelength is a key parameter that determines the axial resolution on the monitor.^15^ Frequency is the key parameter that determines the depth of penetration of ultrasound.

### 1.8 Production of sound and the pulse‐echo principle

**FIGURE 11:**
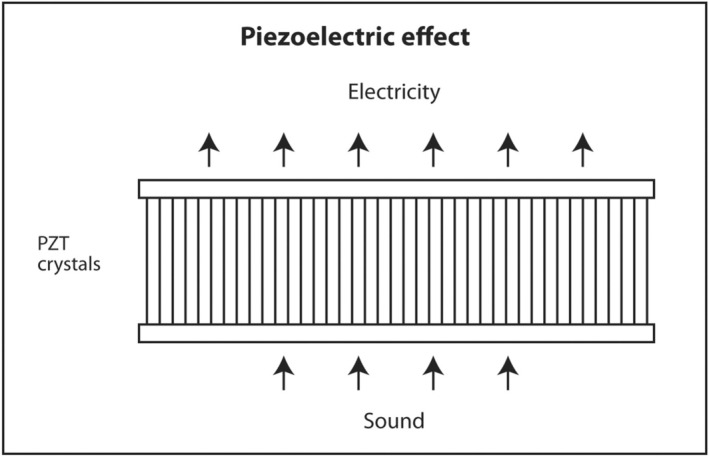
When sound impacts PZT crystals, the energy is converted into electricity.

**FIGURE 12:**
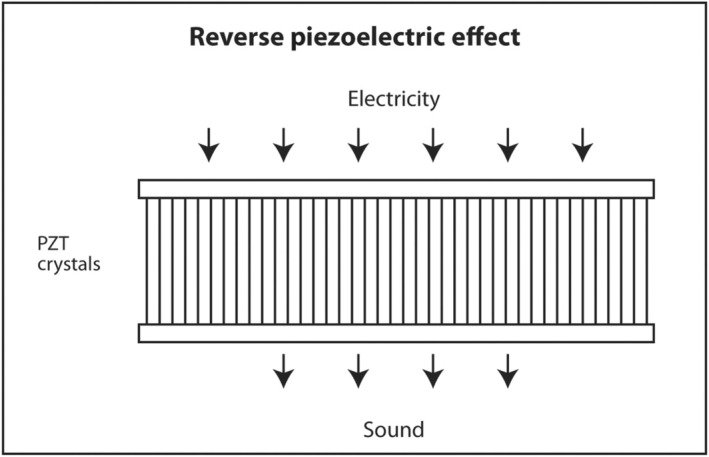
When electricity is applied to PZT crystals, sound is produced.

The first law of thermodynamics states that energy can be converted from one form to another but cannot be created or destroyed. A well‐known example of interconversion of energy is the photoelectric effect. When light of sufficiently high energy (frequency) strikes certain metals, electrons are released from the metal and produce electricity. Conversely, when high‐voltage electricity passes through thin metal filaments such as a tungsten light bulb, light and heat are produced. There is a similar effect for sound. When sound hits certain crystals, such as quartz, tourmaline or Rochelle salts, electrons are released and produce electricity. This is called the piezoelectric effect (Figure 11). When electricity is sent into these crystals, sound is produced. This is called the reverse piezoelectric effect (Figure 12). Modern ultrasound machines use lead zirconate titanate (PZT) crystals to produce sound. This synthetic crystal was chosen rather than natural crystals because it has an efficient coupling coefficient between electricity and sound so less energy is wasted as heat in the interconversion.

**FIGURE 13:**
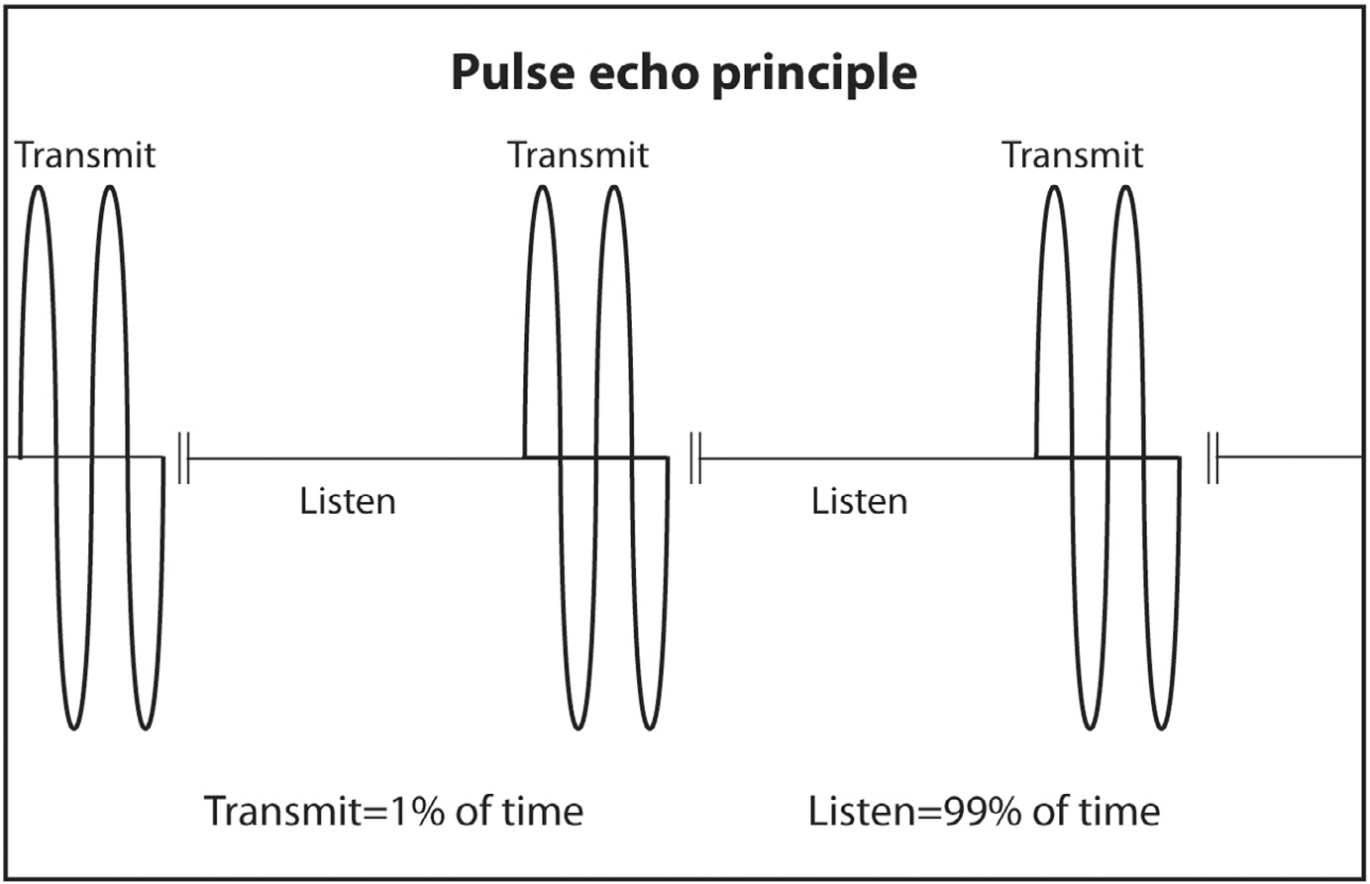
The ultrasound machine sends a short burst of electricity to the PZT crystals. The crystals produce a short pulse of ultrasound typically 2–3 wavelength long. The ultrasound machine then spends a long time waiting for echoes to return. More than 99% of the time is spent waiting for echoes to return. The PZT crystals convert returning echoes into electricity.

**FIGURE 14:**
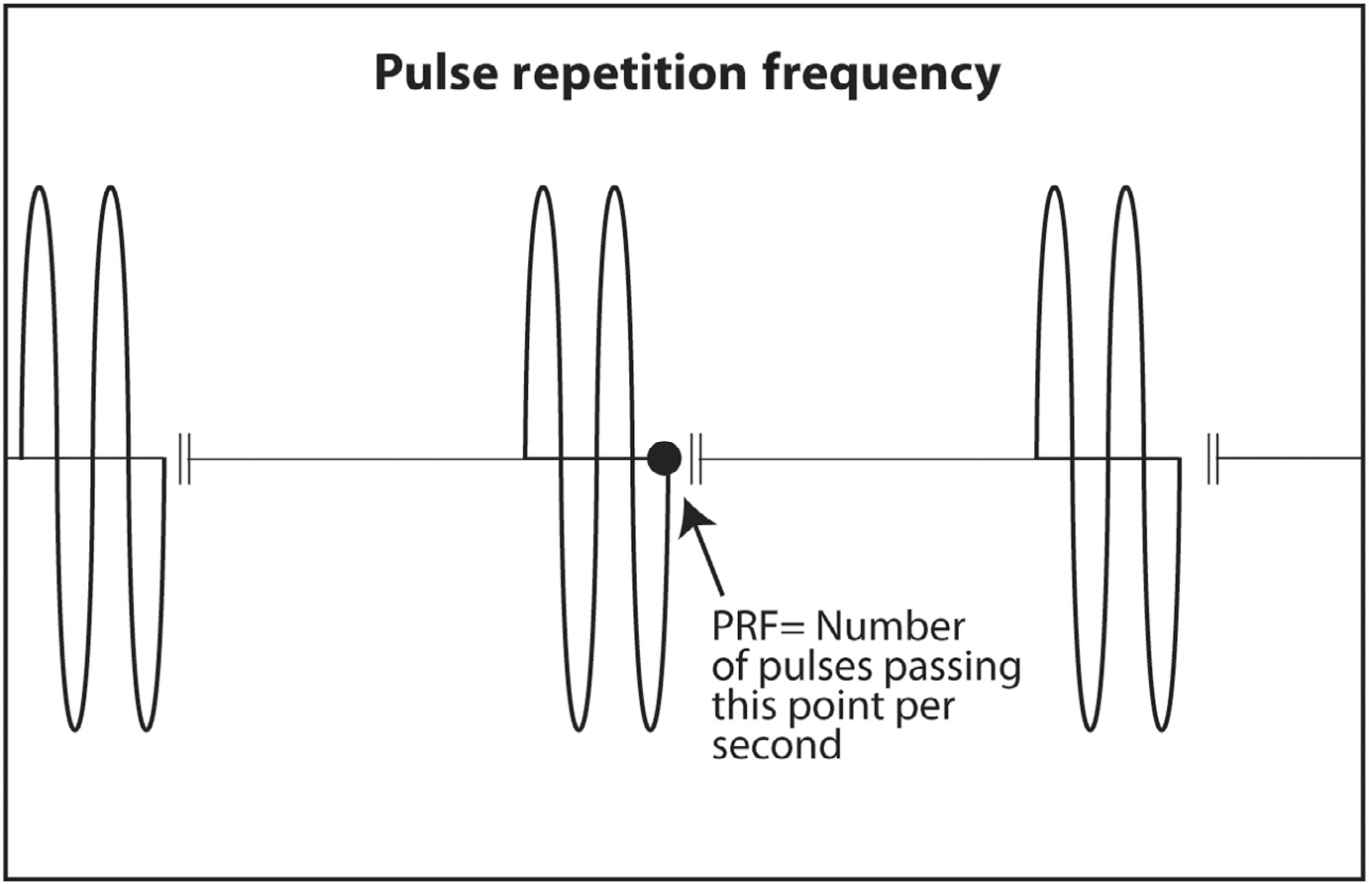
The number of short bursts of sound produced by the ultrasound machine is called the pulse repetition frequency. It is typically 1000–10,000 Hz. In contrast, the frequency of ultrasound produced by linear transducers is 10–20 MHz.

PZT crystals in the transducer both produce sound from electricity and convert returning sound echoes from tissue into electricity. But they cannot do both at the same time. This would be like trying to talk and listen at the same time. In a linear transducer, the type of transducer typically used to scan superficial objects such as thyroid, neck, salivary glands and breast, there are 100–300 thin PZT crystals organized in a linear array. A short burst of electricity is sent into the crystals in a rapid sequential fashion (crystals 1–3, then crystals 4–6, then crystals 7–9, etc.). The crystals produce a short pulse of sound that is transmitted into the body (Figure 13). The ultrasound machine then waits for echoes to return. The returning echoes are converted back into electricity by these same crystals. The electrical signals produced by the returning echoes are amplified and through a series of engineering manipulations converted into an image on the ultrasound screen. The images produced are a function of which crystals received the returning echoes (shape of the objects scanned in the horizontal axis on the ultrasound screen), strength of the returning echoes (brightness of objects on the screen) and timing of the returning echoes (depth in the vertical axis on the ultrasound screen). The number of pulses and subsequent returning echoes produced per second is called the pulse repetition frequency (PRF) (Figure 14). It is usually 1000–10,000 times per second. It is distinct from the transmitted frequency of ultrasound, which is typically 7.5–24 MHz in a linear transducer. The time duration for a single ultrasound pulse and the waiting period for the corresponding echo to return are not the same. A typical pulse is very short and about 2–3 wavelengths long. At a transmitted frequency of 10 MHz, the duration of the pulse would be about 0.2–0.3 μs. The waiting period is two orders of magnitude longer. The transducer spends <1% of the time producing ultrasound and more than 99% of the time waiting for echoes to return. The fraction of time an ultrasound machine spends producing sound compared to the time duration of the pulse‐echo is called duty factor. The duty factor during a typical scan B‐mode scan for anatomy is <1%.

The clinical implication of PRF is twofold. Since the ultrasound machine must wait for echoes to return before transmitting another sound pulse, the depth of the objects being scanned and the speed of sound in the body limit PRF. The speed of sound in the body cannot be changed because speed is an inherent property of the medium. Deeper objects take more time for sound to reach them and for echoes to return. If PRF were too high while scanning deep objects, the new outgoing sound pulse would clash with a previous returning echo. Ultrasound machines are designed so that does not occur when scanning deep anatomy. However, the frame rate (refresh rate on the ultrasound screen) may be lower when scanning deep objects than when scanning superficial objects because the machine must wait longer for deep echoes to return. That is, temporal resolution is worse when scanning deep objects. Temporal resolution is important when performing real‐time ultrasound‐guided biopsies. A scan is considered real time when the frame rate is 15 fps or faster. Therefore, during an ultrasound‐guided biopsy, the depth on the screen should be adjusted so the target fills much of the screen. This allows the target to be more easily seen, decreases unneeded depth, increases PRF and increases frame rate. The second clinical importance of PRF is the direction of blood flow in arteries. If PRF is low because the sonographer sets it too low or a deep object is being scanned, the ultrasound machine may give a false reverse direction of blood flow on the ultrasound screen. This is called aliasing. It is of no importance to interventionalists performing a biopsy. The presence and location of large blood vessels to be avoided during a biopsy are important but the actual direction of blood flow is irrelevant.

### 1.9 Linear transducers

**FIGURE 15:**
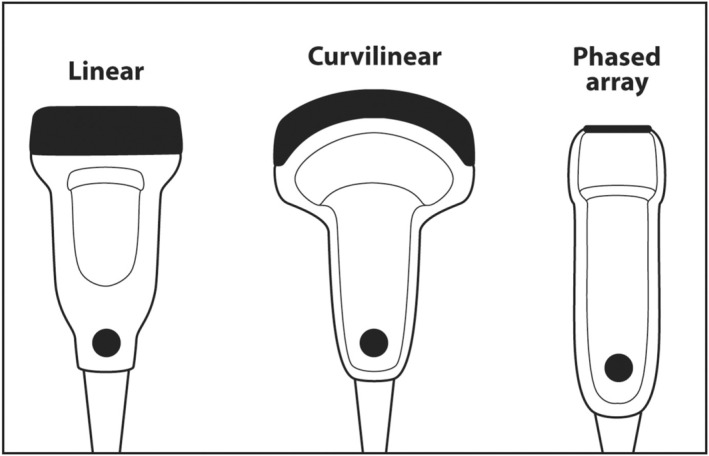
Linear transducers have a large rectangular footprint. They have the thinnest PZT crystals and produce ultrasound with frequencies of 7.5–24 MHz. They are used to scan superficial targets such as thyroid, head and neck, salivary glands, breast, axilla and inguinal. Curvilinear (convex) transducers have a curved footprint. They have thicker PZT crystals and produce ultrasound in the range of 3–7 MHz. They are used to scan deeper structures such as abdomen, pelvis, pregnant uterus and liver. Phased array (sector) transducers have a small square footprint. They have the thickest PZT crystals and produce ultrasound in the 2.5–5 MHz. range. They are used to scan the deepest structures such as heart, liver, aorta and vena cava. The small footprint allows them to be placed between ribs so that a rib shadow does not appear on the ultrasound monitor.

When scanning superficial structures such as thyroid, head and neck, salivary glands, axilla and breast, a linear transducer should be chosen.^16^ Linear transducers have the thinnest PZT crystals and produce the highest frequency ultrasound.^17^ High‐frequency sound results in the best axial resolution on the vertical axis of the ultrasound screen. Other transducers, such as curvilinear (convex) and sector (phased array), have thicker crystals and produce lower frequency sound that is useful for scanning deep anatomy such as the abdomen, liver, pelvis and heart (Figure 15).

**FIGURE 16:**
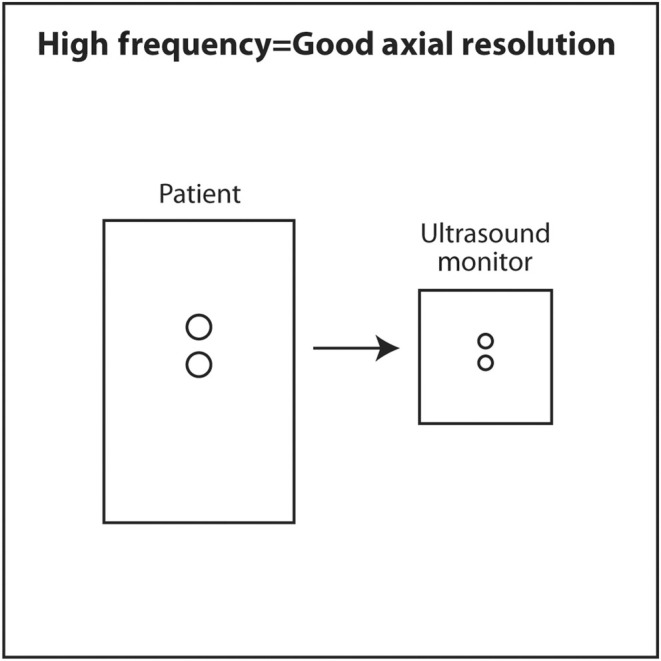
High‐frequency sound results in short wavelength, short SPL and good axial resolution. For geometric reasons, axial resolution is equal to ½ SPL.

**FIGURE 17:**
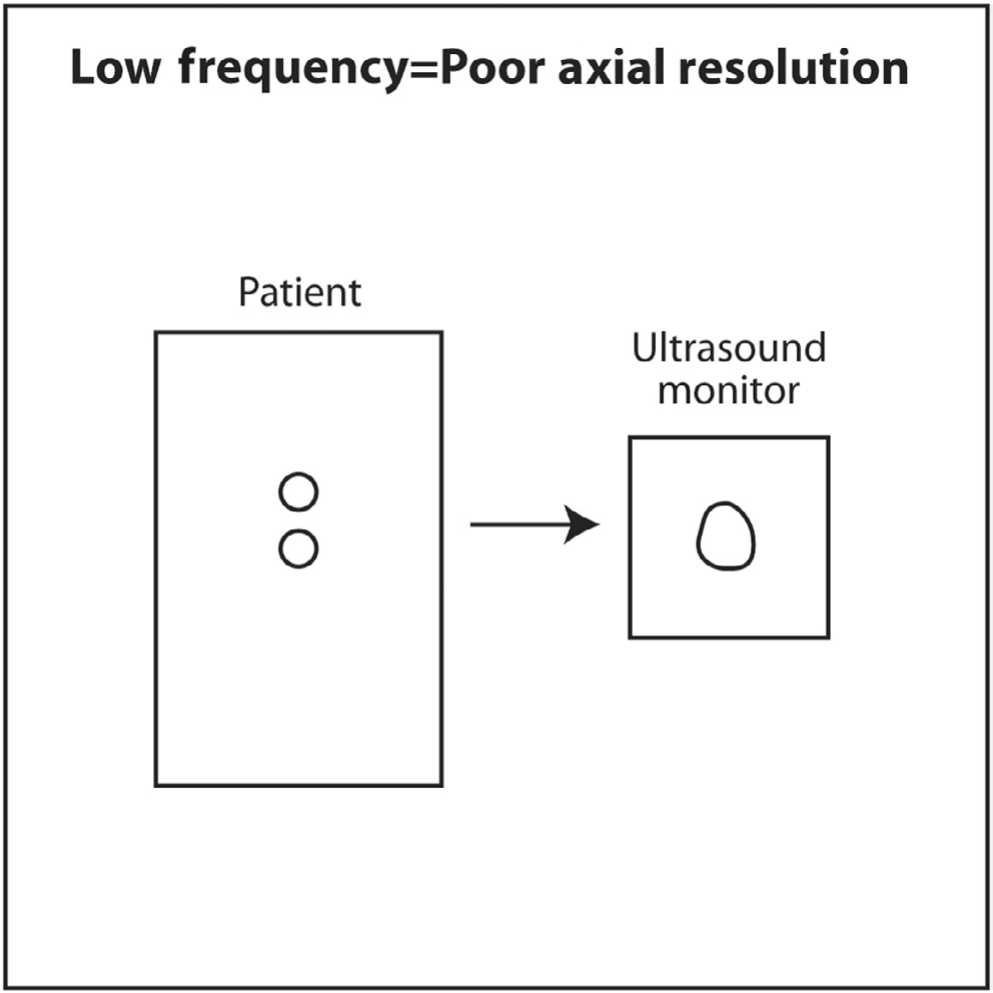
Low‐frequency sound results in long wavelength, long SPL and poor axial resolution. Tissue penetration is greater with low‐frequency sound.

If the frequency of ultrasound produced by a crystal depends on its thickness, how can a single transducer produce sound of various frequencies? When using a particular transducer, three different frequencies can typically be chosen by the touch of a button. The answer lies in the material behind the PZT crystals. Behind the PZT crystals is backing material composed of tungsten and epoxy. This material serves two purposes. It dampens sound after the PZT crystals are stimulated by an electric current so that a short pulse of sound is produced that is only 2–3 waves long rather than continuous. The length in space of these 2–3 waves is called spatial pulse length (SPL). For geometric reasons, resolution in the axial plane is ½ × SPL.^18^ Axial resolution is the closest distance to two objects that can be seen on the vertical axis of the ultrasound screen and still be distinguished as two separate objects. The shorter the SPL, the better is axial resolution (Figure 16). This can be accomplished by increasing frequency, which shortens wavelength. Shorter wavelength shortens SPL. Theoretically, SPL can be decreased by decreasing the number of waves in a pulse. However, the number of waves in a pulse is usually determined by the manufacturer and cannot be changed. Lower frequency results in longer wavelength, longer SPL and worse axial resolution (Figure 17).

The second effect of backing material is to broaden the frequency spectrum of sound produced by the crystals. Without backing material, a 10 MHz transducer would produce only sound continuously at 10 MHz. With the backing material, the sound is dampened into a shorted pulse with sound at frequencies at perhaps 5–15 MHz with the highest amplitude sound at 10 MHz. The machine can filter the sound produced so that the sonographer or interventionalist can select a central frequency of 8, 10 or 12 MHz for scanning. Some machines will designate three different frequency modes rather than the numerical central frequency. For example, resolution mode uses the highest frequency available in a particular transducer. General mode uses an intermediate frequency. Penetration mode uses the lowest frequency.

### 1.10 Echo production and amplification

Images are created when echoes return from the body are converted into electrical signals and then into visible light on the ultrasound monitor. Echoes are produced at the interface between two objects or two areas in the same object that have different impedances. Impedance is the resistance to the transmission of sound. It is analogous to resistance in DC electrical circuits governed by Ohm's law or impedance in AC circuits. The impedance of matter to sound is *Z* = (*ρ*) (*c*) where *Z* = acoustic impedance in rayls, *ρ* = density in kilogram per cubic meter and *c* = speed of sound in meters per second. The typical impedance of human tissue is 1.4 million rayls. The greater the differences in impedance between two different tissues, the stronger the echo produced. A cyst that contains few or no cells and protein is essentially water. Water has uniform impedance. Therefore, a cyst appears anechoic (completely black) on ultrasound because no echoes arise from within it. In contrast, the interface between a rim calcification around a fibroadenoma or the pleura between chest wall and the lung is bright white. Calcium has much higher impedance than a soft tissue mass. The chest wall has much higher impedance than air in the lung. Therefore, strong echoes are produced at these interfaces. Even at the specular (mirror‐like) reflectors, only about 1% of the sound beam is reflected as an echo.

The texture of tissues seen on ultrasound is the result of complex interaction of sound with matter resulting in strong echoes from specular reflectors and diffuse scatter of sound from irregular reflectors. Masses have different echotexture than normal tissue and stand out on the ultrasound monitor.

Echoes arising from tissue are attenuated (weakened) before returning to the transducer. The effect is more pronounced for deep structures because the transmitted sound beam travels through more tissue and the returning echoes must travel through the same amount of tissue. As previously mentioned, most of the sound is converted to heat but some of it is scattered. The decrease in power of the sound is so pronounced that it is measured on a logarithmic scale called decibels (dB). This scale is similar to the more familiar Richter scale for measuring the power of an earthquake. A 3 dB decrease in power is a 50% decrease. A 10 dB decrease in power is a 10‐fold decrease. A 20 dB decrease in power is a 100‐fold decrease. A 30 dB decrease in power is a 1000‐fold decrease. The attenuation coefficient for soft tissue is about 0.5 dB/MHz‐cm. Therefore, a 10 MHz beam that traverses 4 cm of soft tissue encounters a specular reflector‐like bone and returns an echo to the transducer will have a 60 dB decrease in power or 1/1,000,000 of the original strength of the transmitted sound beam.

In order for the weak returning echoes to produce an image visible on the ultrasound monitor, the weak electrical signals produced by the piezoelectric effect must be greatly amplified. All electric signals produced by the returning echoes are greatly amplified. This is called gain. When the same amount of gain is applied equally to all echoes, it is called overall gain. Gain is also measured in decibels. Late returning echoes from deep structures are even weaker than those from superficial structures because the former must travel though more tissue and are more attenuated. In order to avoid an image that is bright superficially (strong returning echoes) and dark deeply (weak returning echoes), the ultrasound machine will apply more gain to late returning echoes and less to early echoes. This is called time gain compensation. Most of this differential amplification is automatically done by the machine so that an image is uniformly grey at all depths. However, patients are different and the automatic adjustments may not be ideal to produce a uniform image. All modern machines allow the operator to adjust gain (increase or decrease) in the vertical axis to produce a uniform image. Some machines even allow adjustment in the horizontal axis.

### 1.11 Resolution

There are four types of resolution in ultrasound.^19^ Three are spatial and the fourth is temporal. The three spatial dimensions are axial (vertical on the screen), lateral (horizontal on the screen) and elevational or slice thickness (in an out of the plane of the screen). The single temporal dimension is essentially the frame rate or refresh rate of the monitor. The goal of imaging is to maximize spatial resolution (smallest distance between two adjacent objects and still be distinguished as two separate objects) and temporal resolution (fastest frame rate). Unfortunately, these entities are not entirely independent.

Axial resolution has been previously discussed and is resolution along the plane parallel to the ultrasound beam. This is the vertical axis on the monitor screen. Axial resolution is ½ × SPL. The shorter the SPL, the better the axial resolution. Since frequency determines wavelength and thus SPL, ultrasound scans should generally start at the highest frequency to obtain the best axial resolution. Using a scan frequency of 10 MHz, assuming the speed of sound is 1.54 mm/μs, and a pulse that is 2 wavelength long, the limit of axial resolution would be ½ × 0.308 = 0.154 mm. In reality, the numerical value could be higher (worse resolution) depending on the on the electronics of image production and resolution on the monitor. Axial resolution does not change very much with increasing depth. The clinical implication of the basic science underlying axial resolution is that an ultrasound scan should start by choosing the appropriate type of transducer for the area to be scanned and starting the scan at the highest available frequency. For superficial structures typically biopsied by interventional cytopathologists, such as thyroid, head and neck, salivary glands, breast and lymph nodes, the best type of transducer to use is linear small parts. Linear small part transducers have the thinnest PZT crystals and produce sound of the highest frequency. The scan should start at the highest available frequency to maximize axial resolution. If the image is too dark, especially in the deepest regions, overall gain and time gain compensation should be increased to brighten the image. If these adjustments fail, a lower scan frequency should be used. If a lower frequency fails to produce an acceptable image, a different linear transducer with a lower frequent range or a curvilinear transducer should be used.

**FIGURE 18:**
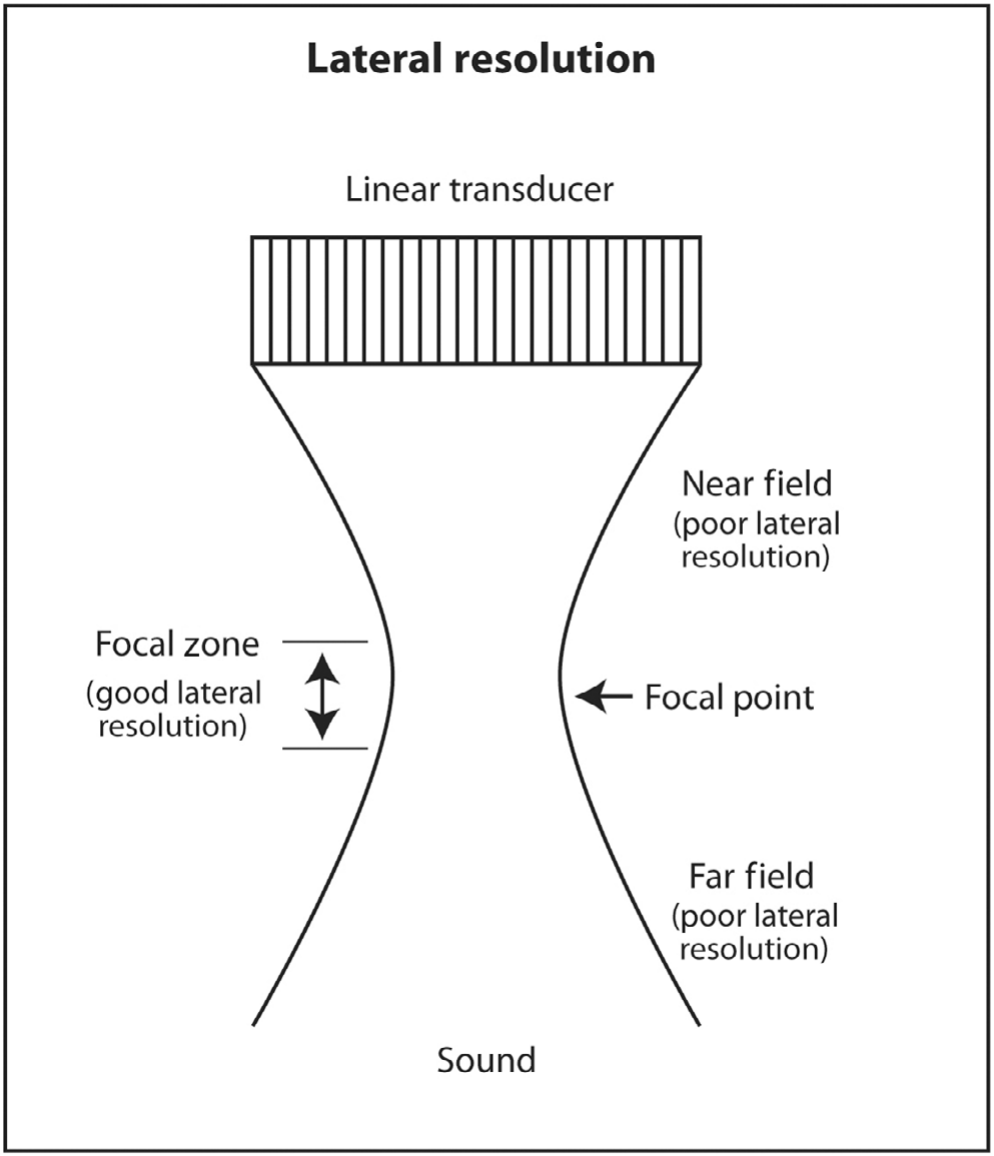
According to Huygens' Principle, the sound waves from the PZT crystals act as point sources of sound. The sound waves combine by constructive and destructive interference to produce an hourglass‐like beam of sound.

**FIGURE 19:**
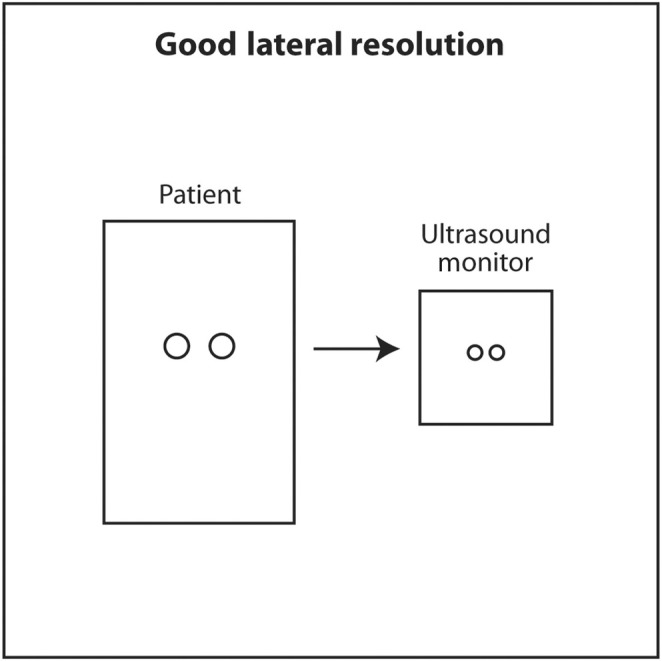
Lateral resolution is best where the beam is thinnest at or near the focal point. In terms of distance, lateral resolution is approximately the width of the beam at the corresponding depth.

**FIGURE 20:**
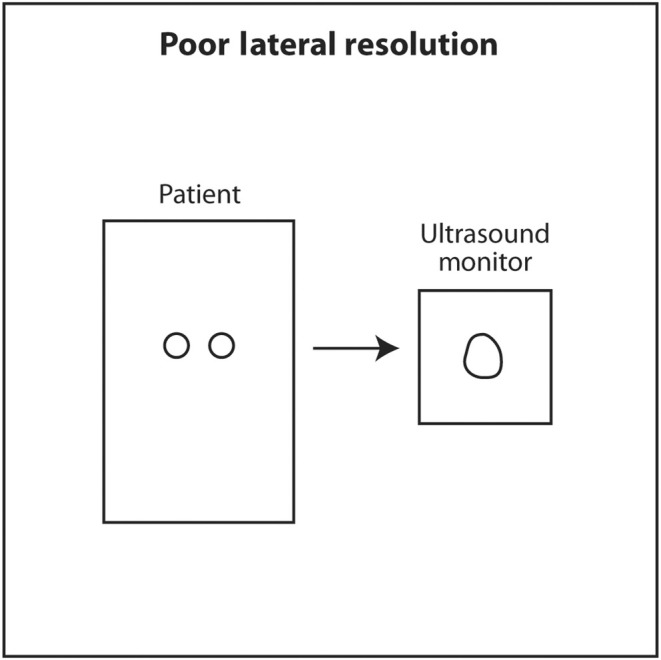
Lateral resolution is worse in the near field or far field because the sound beam is wide.

**FIGURE 21:**
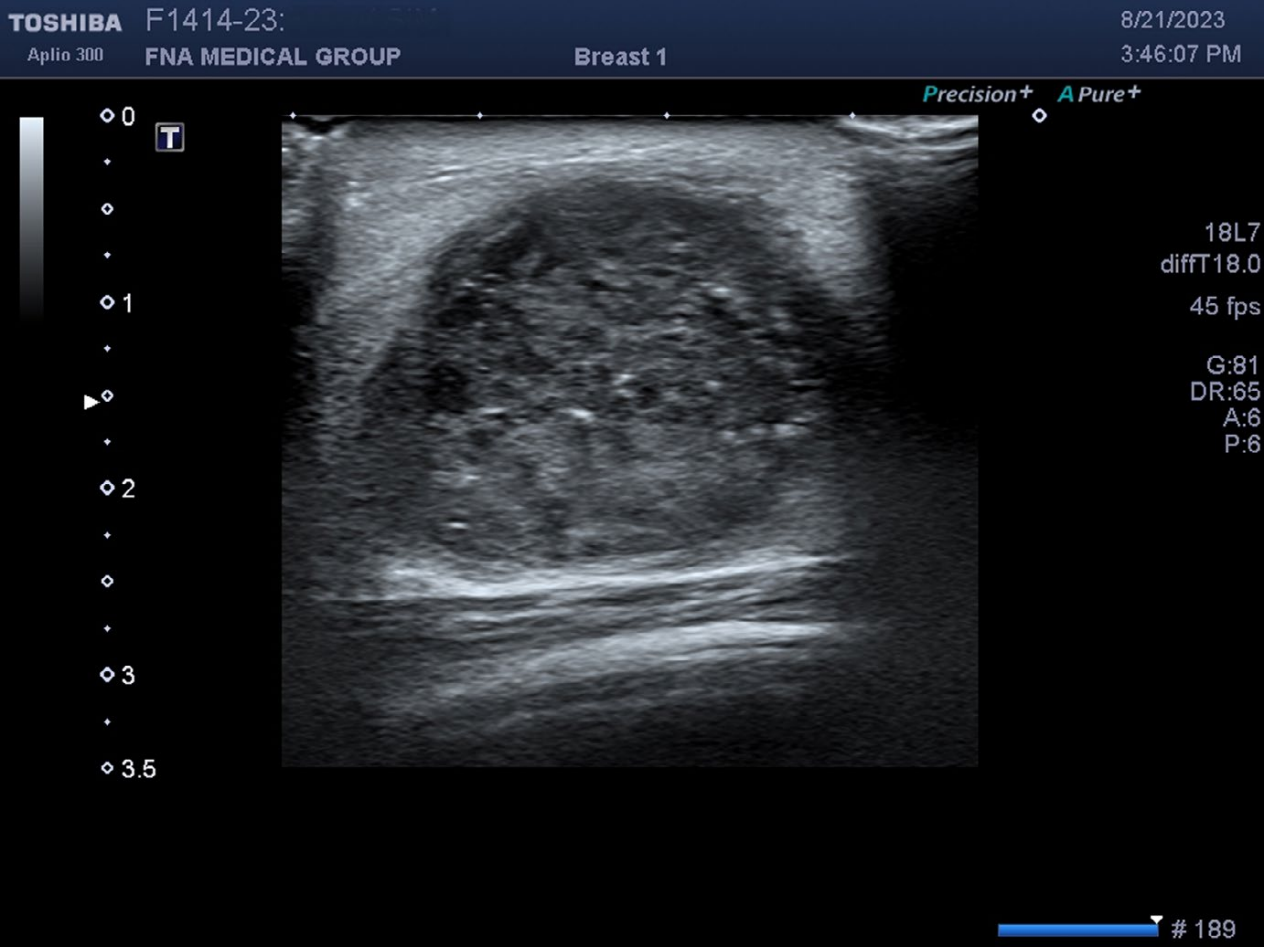
In this malignant breast mass, the focal point (rightwards filled arrow on the left side of the screen) is placed correctly near the middle of the mass at 1.5 cm depth. The echoes inside the mass are sharp.

**FIGURE 22:**
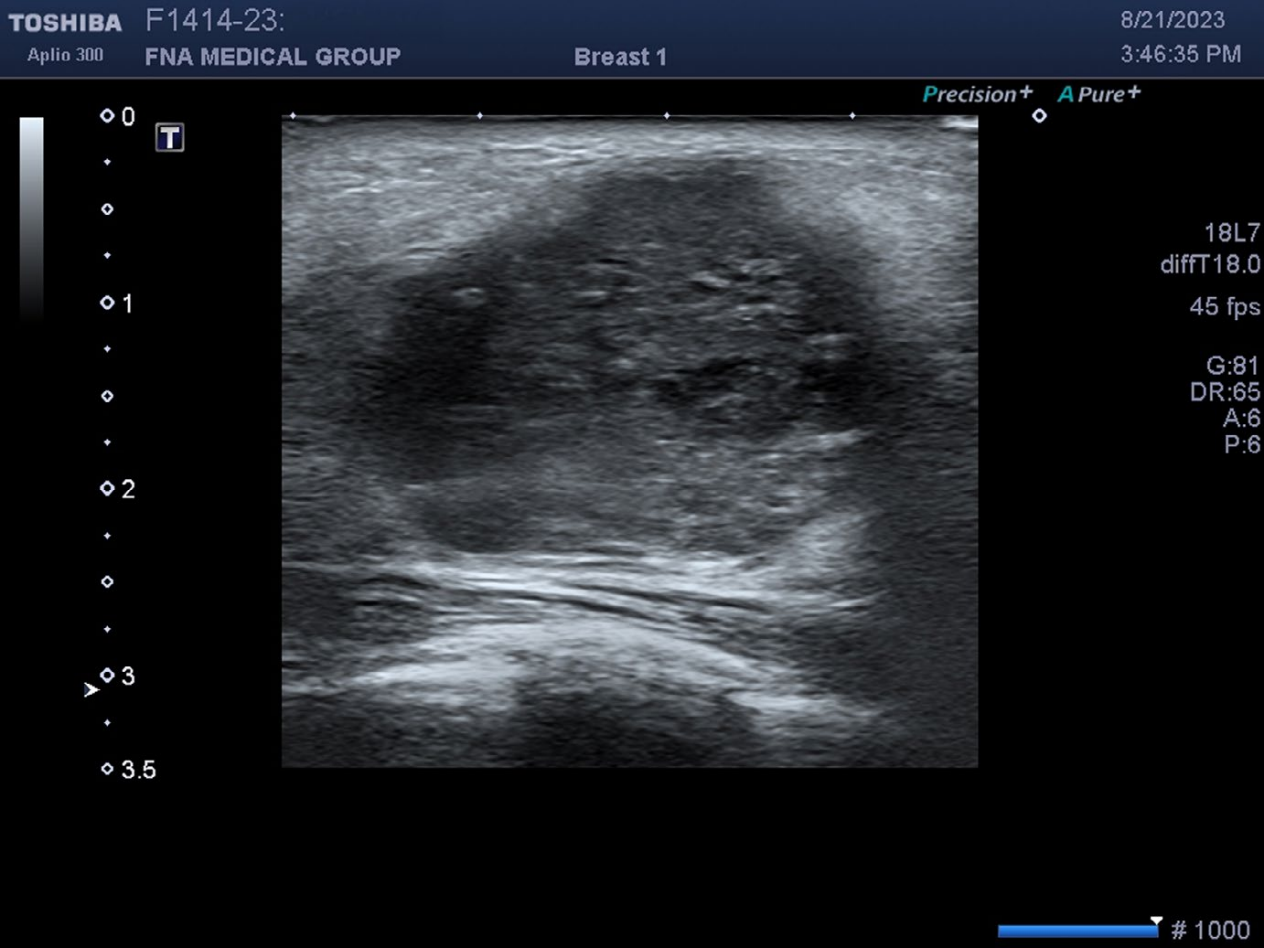
This is the same mass as in Figure 22 but the focus is incorrectly placed too deep at 3 cm (rightward filled arrow on left side of screen). The echoes in the mass now appear blurred.

Lateral resolution is the ability of ultrasound to distinguish between two objects close to each other perpendicular to the direction of the incoming ultrasound beam. This corresponds to the horizontal axis (left and right) on the monitor. Lateral resolution depends on the width of the ultrasound beam at the depth of the object of interest. The thinner the ultrasound beam, the better the lateral resolution. The actual lateral resolution in terms of distance is approximately the width of the ultrasound beam at different depths. Ultrasound emanating from the transducer is wide, narrows to a thin point called the focal point, and then widens again. The shape of the beam is like an hourglass (Figure 18). This is called Huygens' Principle. The region just proximal and distal to the focal point where the beam is relatively narrow is called the focal zone. Lateral resolution is best in the focal zone (Figure 19). The region near the transducer is called the near field or Fresnel zone. The region distal to the focal zone is called the far field or Fraunhofer zone. Lateral resolution is worse in the near and far field compared to the focal zone because the beam is wide (Figure 20). The distance of the focus from the transducer depends on the frequency of the transducer and the diameter of the PZT crystal. However, in modern ultrasound machines, the location of the focal point and thus the focal region can be changed by changing the sequence of electrical signals sent to the PZT crystals. The sonographer or interventionalist can move the focal point by the touch of a button on the ultrasound machine. The clinical implication is that the focal point should be moved so that it is located in the middle or lower half of the object of interest or target for biopsy (Figures 21 and 22). This will insure that the target is best seen in the horizontal or lateral plane. In general, lateral resolution is not as good as axial resolution.

The third dimension of spatial resolution is elevational or slice thickness resolution. This is resolution in the plane in and out of the monitor screen. Since the anatomy being scanned is three‐dimensional and the ultrasound monitor is two‐dimensional, the display is basically a projection of a three‐dimensional object onto a 2D screen. The thinner the slice in the third dimension, the clearer an object will appear on the screen. Similar to lateral resolution, the ultrasound beam in this third dimension has an hourglass shape with a focal point and focal zone. Most linear small part transducers at 1D have 100–300 thin PZT crystals arranged in a linear array. The location of focal point in 1D transducers cannot be changed by the sonographer or interventionalist. These transducers have an acoustic lens inside that places the focal point in the elevational plane about 1.5 cm from the face of the transducer. The reason for placing the focal point at this depth in a linear transducer is that most targets will be superficial and located at about this depth when using a linear transducer. If a deeper object, perhaps at 5 cm depth, was scanned using a linear small part transducer (assuming it is still visible while being scanned at a high frequency), it might appear blurry because the elevation focus is located much more superficially. In a lower frequency 1D transducer, such as a curvilinear (convex) transducer, the acoustic lens will place the focal point in the elevational plane at about 4.5 cm from the face of the transducer. The reason for this location is that curvilinear transducers are used to scan deeper anatomy, such as abdomen or pelvis. The elevational focus at 4.5 cm depth will be closer to the actual depth of the objects of interest. The clinical implication for sonographers and interventionalists is to select a linear small part transducer when scanning superficial objects and a curvilinear transducer when scanning deeper objects. Linear transducers transit a higher frequency to maximize axial resolution and the acoustic lens place the fixed elevational focus superficially. When scanning deeper objects, a curvilinear transducer should be selected. The lower transmitted frequency allows better sound penetration. The deeper location of the fixed elevational focus is closer to the depth of deeper objects of interest.

Temporal resolution is last dimension of resolution. It is essentially the refresh rate of images on the ultrasound monitor. The goal of temporal resolution is to maximize the refresh rate, especially during a biopsy because real‐time location of the needle is important. Much of the refresh rate depends on the electronics of the ultrasound machine. This is analogous to the clock speed of the chip in a computer. Faster computers have a chip with faster clock speed. The ultrasound machine is a specialized computer. Modern ultrasound machines have faster temporal resolution than older ones. There are some aspects of temporal resolution that can be controlled by the sonographer or interventionalist and others that cannot be controlled by him or her. According to the pulse‐echo principle, the ultrasound machine transmits sound into a patient and waits for the corresponding echo to return. Since the speed of sound in matter is dependent on the stiffness and density of the medium and not the frequency or power of the transmitted sound wave, it is a fixed acoustic parameter that limits temporal resolution. Theoretically, if the speed of sound could be increased, temporal resolution could be increased but this is not possible.

The depth of the ultrasound scan can be controlled by the sonographer or interventionalist. When deep objects are scanned, the ultrasound machine must wait longer for echoes to return. This longer wait negatively impacts temporal resolution and decreases the refresh rate. On the other hand, during a diagnostic ultrasound scan, the depth must be set deep enough so that deep pathology is not missed. For example, a diagnostic breast ultrasound should scan all the way down to the pleura. If the depth is not set deep enough and breast tissue is present below the deep margin on the ultrasound monitor, a tumour deep to the field of view could be missed. The clinical implication is once the location of the object to be biopsied is established, the scanning depth can be adjusted so that the target appears larger. During a biopsy, tissue much deeper than the target is of no interest. This adjustment will make biopsy easier since the target appears larger and improves temporal resolution.

**FIGURE 23:**
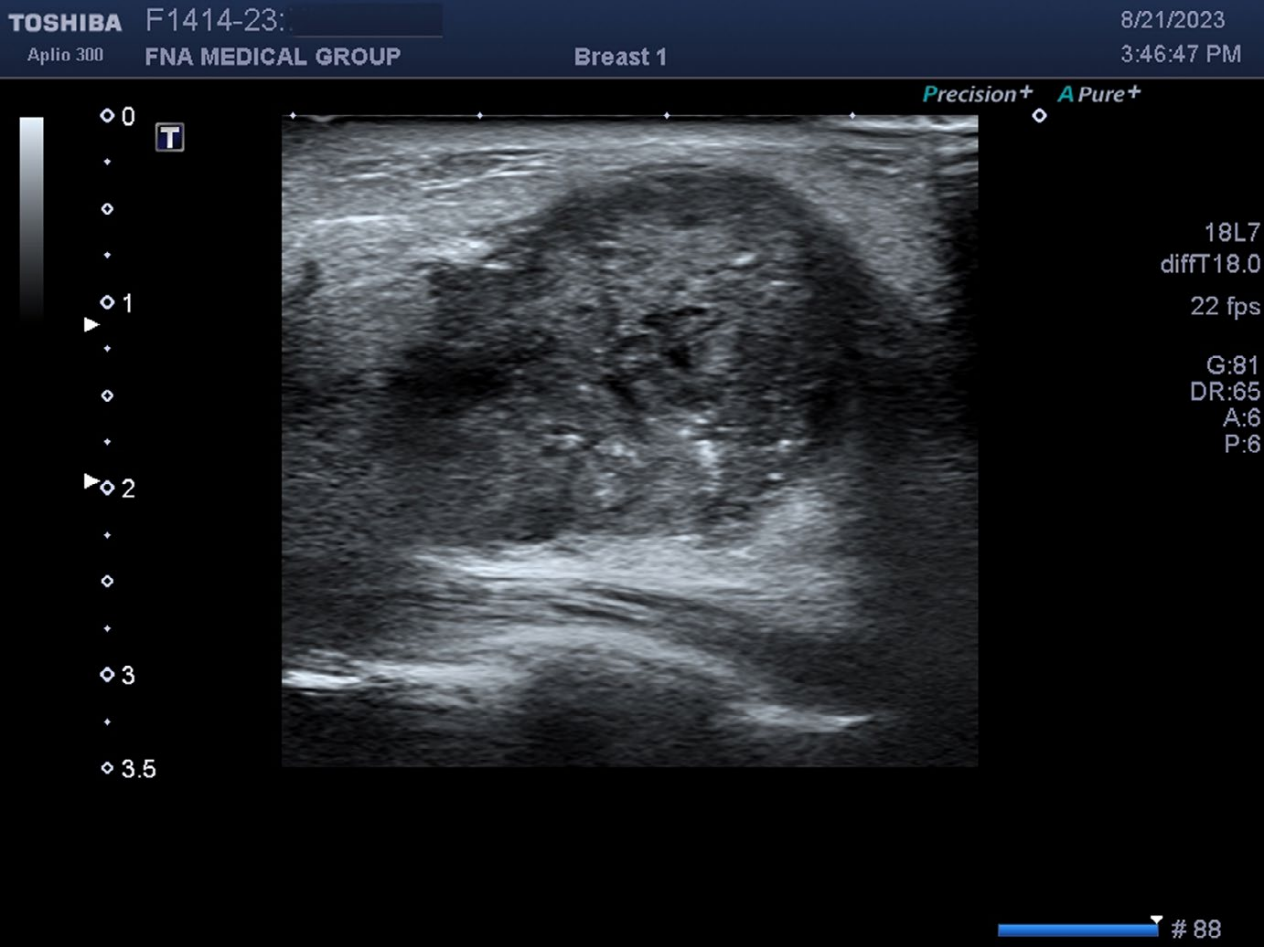
This is the same image as in Figure 22 but two foci are now used to produce the image. They are located at 1 and 2 cm depth (rightwards filled arrow on the left side of the screen). In Figure 21, the frame rate is 45 fps (right‐hand side of screen). In this image, the frame rate is 22 fps (right‐hand side of screen). Additional foci improve lateral resolution but degrade temporal resolution.

Another determinant of temporal resolution is the number of foci. As previously discussed, the location of the lateral focus can be adjusted up or down on the ultrasound monitor by the sonographer or interventionalist to improve lateral resolution at the depth of the object of interest. The numbers of foci can also be changed from 1 to as many as 4 depending on the machine. While increased number of foci will improve lateral resolution at multiple depths, it will worsen temporal resolution (Figure 23). The ultrasound machine needs more time to reconstruct the data from multiple foci to produce a single image on the monitor. The clinical implication of number of foci and temporal resolution is that sonographers and radiologists are concerned about detecting pathology on a diagnostic scan. They do not want to miss small or subtle pathology. Therefore, multiple foci are often used to maximize spatial resolution. Temporal resolution is less important. Interventionalists already know the location of the target to be biopsied that was discovered by the sonographer and radiologist. Real‐time accuracy of needle placement is more important. Therefore, typically only a single focus is used during a biopsy to maximize temporal resolution.

### 1.12 Doppler imaging

The last important topic in ultrasound fundamentals is Doppler imaging. The Doppler Effect is familiar to most people. It is the change in the frequency of sound when the source of sound or receiver of sound is moving with respect to each other. A familiar example is the frequency of sound from an ambulance changes as it is moving towards or away from the listener. In diagnostic ultrasound, the only object moving fast enough to cause a Doppler shift in frequency in returning echoes is blood in blood vessels. The ultrasound machine can detect a shift in the average frequency in the frequency spectrum (frequency of sound on the *x*‐axis and amplitude of sound on the *y*‐axis) of the transmitted ultrasound compared to the returning echoes.^20^ This shift in average frequency gives information on the direction of blood flow and its velocity. It can be displayed as a colour flow scan with flow information in colour placed on top of an anatomic (B‐mode) scan. This basically shows the location of blood vessels and the direction of blood flow. It can also be displayed digitally showing average and peak velocity of blood flow during a spectral Doppler scan.

A similar concept is power Doppler that detects motion based on the total amplitude of objects that are moving. Power Doppler displays motion and in advanced machines, direction of flow, but since it detects total amplitude of a phase shift and not frequency shift, it cannot calculate the velocity of blood flow. Power Doppler is more sensitive and less angle dependent than colour Doppler. The clinical implications for the interventionalist are the presence of blood vessels, location of blood vessels and distribution of vessels seen. Increased vascularity and abnormal blood vessel distribution may suggest a malignant neoplasm. Sometimes subtle masses can be outlined using Doppler. Large blood vessels should be avoided when performing a biopsy. The direction of blood flow and its velocity are important to vascular surgeons but not to interventional cytopathologists. Therefore, power Doppler is more often used by interventionalists than colour Doppler.

**FIGURE 24:**
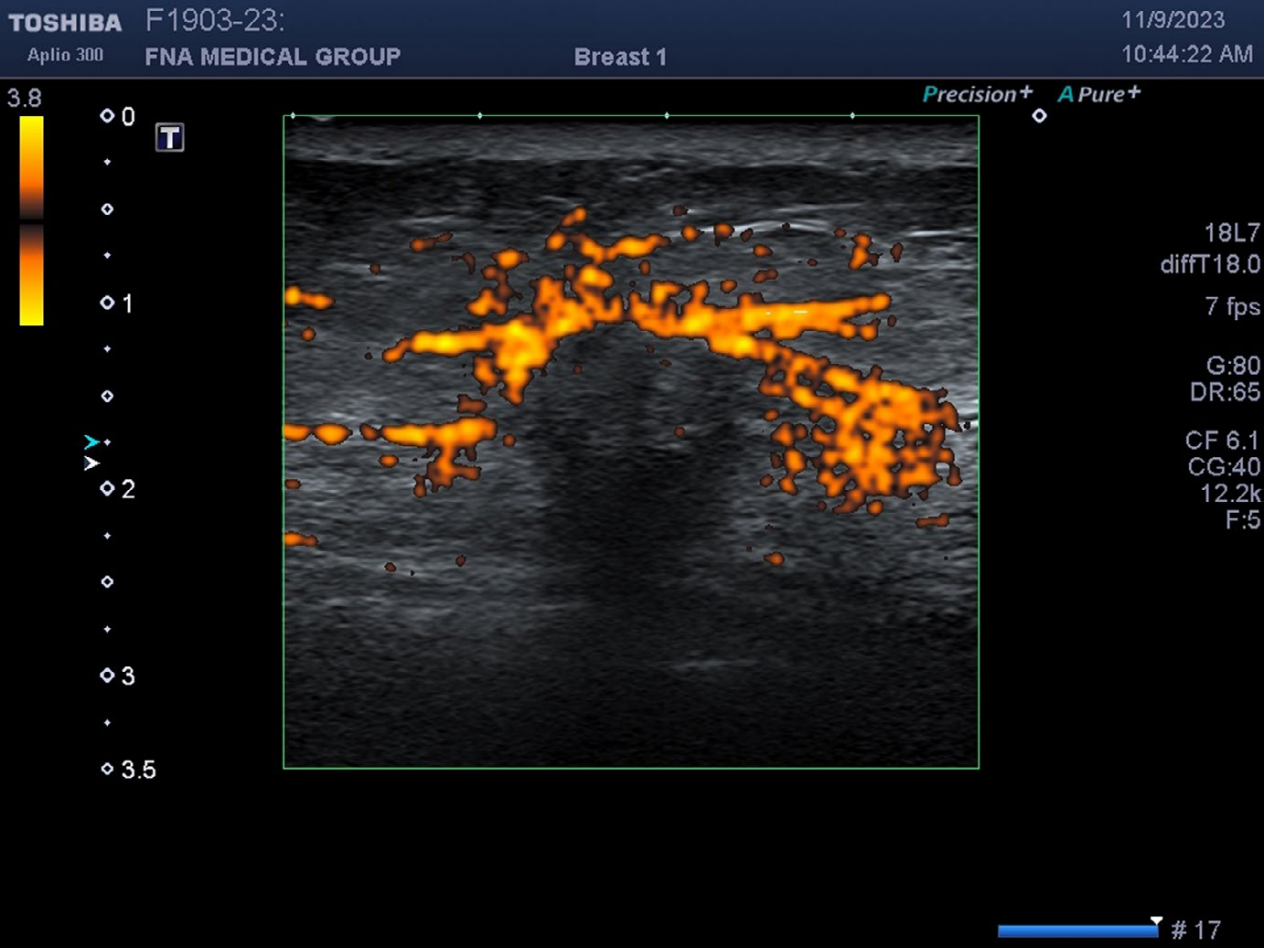
During power Doppler vocal fremitus, the patient says ‘eeeeee…’ loudly with power Doppler turned on. The tissue surrounding the mass vibrates and produces a Doppler signal. The mass does not vibrate and is outlined by an orange flash. This could help identify subtle breast masses.

A useful technique to identify subtle or isoechoic breast masses is power Doppler vocal fremitus.^21^ In this technique, the patient is asked to say ‘eeeeeee’ loudly for several seconds while a breast mass is being scanned with power Doppler turned on. The vocalization will cause normal tissue to flash orange due to tissue motion. However, a mass will not vibrate like surrounding normal breast tissue and will not appear orange. The orange flash of surrounding tissue will outline the mass (Figure 24).

Acquisition of a colour Doppler or power Doppler scan requires more time for the ultrasound machine to produce an image. Therefore, temporal resolution tends to be very poor (slow frame rate) while scanning with colour or power Doppler. Real‐time biopsy is typically performed in B‐mode with colour and power Doppler turned off because the frame rate is too slow when Doppler is applied. Also, motion of the biopsy needle during an interventional procedure will cause flash artefact that obscure the anatomy and target making biopsy more difficult.

## 2 CONCLUSION

The principles underling diagnostic ultrasound are very different from those of other imaging modalities. It does not rely on X‐rays penetrating tissue, energy from protons as they return to a lower energy state in a magnetic field, positron emission or gamma ray emission. Ultrasound produces images from sound echoes reflected from tissue much like sonar or radar. Interventional cytopathologists need only a very basic understanding of ultrasound physics and instrumentation to be able to produce an acceptable image and perform a biopsy. The most important concepts are frequency, attenuation, overall gain, time‐gain compensation, focus, spatial resolution, temporal resolution and Doppler. The goal of intervention is to use ultrasound guidance to help adequately sample a mass so an accurate pathologic diagnosis can be made.

## AUTHOR CONTRIBUTIONS

Single‐author paper with all work done by the author.

## CONFLICT OF INTEREST STATEMENT

The author has no conflict of interest to declare.

## ETHICS STATEMENT

This study was approved by Fine Needle Aspiration Medical Group.

## Data Availability

Data sharing is not applicable to this article as no new data were created or analysed in this article.
